# Structural Ordering and Polarizable Al^3+^ Solvation Induced via Amphiphilic Zwitterion for Highly‐Reversible Zn–Al Alloy Anodes

**DOI:** 10.1002/smll.202514994

**Published:** 2026-04-17

**Authors:** Shengyang Huang, Zuyang Hu, Yilang Liu, Dong Hyun Min, Jun Su Kim, Hongdae Lee, Sida Zhang, Guanyao Wang, Zhipeng Wen, Cheng Chao Li, Ho Seok Park, Qingyun Dou

**Affiliations:** ^1^ School of Chemical Engineering Sungkyunkwan University (SKKU) Suwon Gyeonggi‐do Republic of Korea; ^2^ Guangdong Provincial Key Laboratory of Plant Resources Biorefinery School of Chemical Engineering and Light Industry Guangdong University of Technology Guangzhou China; ^3^ School of Materials Science and Engineering Sun Yat‐Sen University Guangzhou China; ^4^ SKKU Institute of Energy Science and Technology (SIEST) Sungkyunkwan University Suwon Republic of Korea

**Keywords:** aqueous batteries, aluminum‐ion batteries, alloy anodes, self‐assembly

## Abstract

Aqueous aluminum‐ion batteries face significant challenges in achieving stable plating/stripping owing to issues such as hydrogen evolution and corrosion. Although the in situ formation of Zn‐Al alloys via Zn anodes and Al^3+^‐based electrolytes enables a suitable operating potential, this approach is hindered by cycling deactivation caused by uneven deposition and side reactions. Herein, we employ an amphiphilic zwitterion (ZI‐10) as an electrolyte additive to enhance the stability of Zn–Al alloy anodes. Small‐angle X‐ray scattering (SAXS) and sum frequency generation (SFG) spectroscopy reveal that the self‐assembly of ZI‐10 forms ordered aggregates, thereby constructing a dual‐layered electric double layer (EDL) that excludes water from the electrode interface while facilitating reversible plating/stripping. The zwitterionic chelation further induces a polarizable Al^3+^ solvation shell that reduces the energy barrier for charge transfer. Ultimately, Zn–Al||Zn–Al symmetric cells achieve >2500 h of stable operation at 1 mA cm^−2^ and 1 mAh cm^−2^, outperforming the recently reported counterparts. Full cells paired with MnVO cathodes sustain stable cycling even under 10 mg cm^−2^ cathode loading, while Zn–Al||I_2_ full cells further demonstrate exceptional long‐term durability. This work offers insights into the role of amphiphilic zwitterions in tailoring electrolyte nanostructure and interfacial reaction kinetics.

## Graphical abstract

An amphiphilic zwitterion of ZI‐10 is employed as an electrolyte additive to enhance the stability of Zn–Al alloy anodes, thereby constructing a dual‐layered electric double layer through the self‐assembly. The ZI‐10‐induced Al^3+^ solvation reduces charge transfer barriers, enabling over 2500 h of stable cycling and superior performance in full cells.

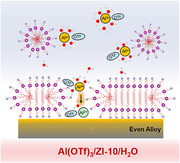

## 1 Introduction

Aqueous multivalent metal‐ion batteries are attractive owing to their safety, low cost, and high ionic conductivity of aqueous electrolytes, coupled with high theoretical capacity of metal anodes [1, 2, 3]. Among multivalent‐metal anodes, aluminum metal stands out as a promising alternative attributable to its natural abundance (8.23% compared to Li's 0.0017%) and high theoretical capacity (2981 mAh g^−1^ compared to Li's 3860 mAh g^−1^) [4, 5, 6]. However, traditional Al‐ion batteries (AIBs) reliably achieve reversible plating/stripping in nonaqueous AlCl_3_‐based ionic liquid electrolytes [7]. The dense oxide layer (Al_2_O_3_), which is spontaneously formed on the Al surface, significantly hinders the Al deposition [8]. Although researchers have developed chemical approaches such as quasi‐solid‐state electrolytes and deep eutectic electrolytes, these methods present technical limitations including complicated processes, compromised ionic conductivity, and potential safety concerns [9, 10, 11].

To overcome these difficulties, current researches on aqueous AIBs have been exploited taking both advantages of aqueous electrolytes and Al metal anodes [12, 13, 14]. However, reversible Al deposition is thermodynamically restricted owing to the Al anode's relatively negative standard reduction potential of −1.662 V versus standard hydrogen electrode, where the hydrogen evolution reaction (HER) occurs in advance of effective Al deposition [15, 16]. The narrow electrochemical stability window of aqueous electrolytes might result in safety risks by gas evolution [17, 18, 19]. Accordingly, various strategies have been actively explored to achieve the reversible and stable operation of aqueous AIBs, including hybrid electrolytes [20, 21], water‐in‐salt electrolytes [22], electrolyte additives [23], and artificial solid‐electrolyte interphases [24]. Among these strategies, the introduction of electrolyte additives is considered as an efficient strategy to improve the electrochemical properties of electrolyte solutions and suppress undesirable side reactions [25]. The rational design and careful selection of such additives can tune the solvation and interfacial structures even with a tiny amount, further improving battery performance [26]. In particular, zwitterionic additives containing both positively and negatively charged groups have recently attracted increasing attention, as their strong dipolar interactions and dual‐functional sites can simultaneously regulate ion solvation structures and stabilize the electrode/electrolyte interface [27, 28]. Despite these intensive researches, the improvement of battery performance by these strategies is far from satisfactory, as their small plating/stripping current density, low capacity and retention, and low mass loading limit practical applications [29, 30].

Recently, researchers have proposed a new approach, where alloying the anode on metal foil in aqueous electrolytes could enable reversible Al^3+^ plating/stripping for a long‐term stability and high efficiency of Al^3+^‐assisted batteries (examples of alloy anodes include Cu–Al, Sn–Al, Zn–Al, Ce–Al, etc.) [31, 32, 33, 34, 35]. Against this backdrop, in situ synthesis techniques have emerged as particularly powerful [36]. For instance, an in situ formed Zn–Al alloy anode—created by reacting Zn foil with Al^3+^ ions in an aqueous electrolyte—demonstrates an extended operational potential range while effectively suppressing HER [37]. Compared to ex situ synthesis, the in situ formation of the Zn–Al alloy is regarded as a more convenient approach that adapts to the microscopic structure of the electrode surface, reducing contact resistance and enhancing charge transfer efficiency [38]. However, the persistent issue of HER in aqueous AIBs remains unresolved, posing ongoing safety risks [23, 39, 40]. This highlights the strong demand on a more in‐depth advancement in electrolyte design for Al‐based alloy anodes. In our previous work, we have successfully enhanced the energy density and lifespan of Zn–Al alloy anodes through the careful design of hybrid electrolytes [41]. However, the use of organic solvents like acetonitrile, with its strong volatility and harmful health effects, has emphasized the need for green, aqueous‐based systems [42, 43]. In addition, several previous studies have explored the incorporation of Al‐related species to stabilize Zn metal anodes via Zn–Al alloy in conventional aqueous zinc ion batteries (ZIBs) [44, 45]. However, these systems are fundamentally based on Zn^2+^ charge carriers and primarily aim to regulate Zn deposition behavior, which distinguishes them from studies focusing on Al^3+^ charge carriers and their associated electrochemical processes.

**FIGURE 1:**
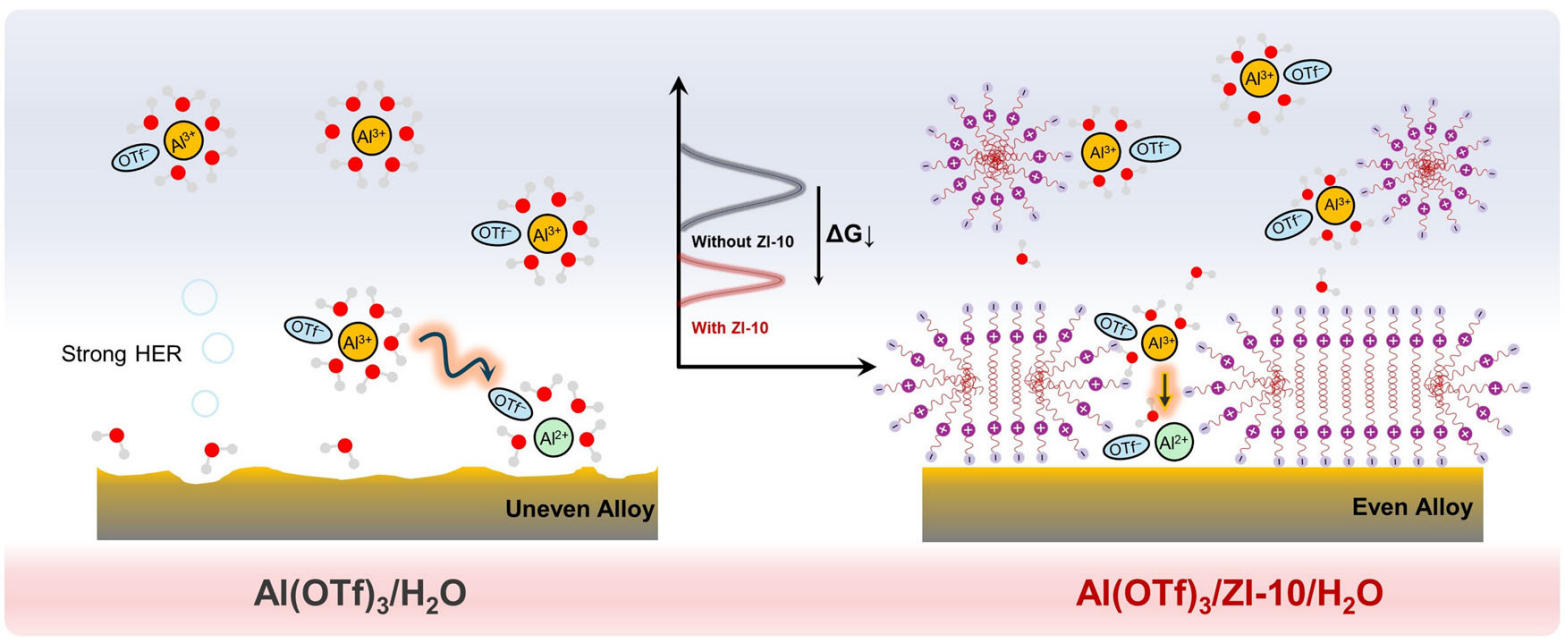
Schematic illustration of the self‐assembly and interfacial modification of ZI‐10 for Zn–Al alloy anodes. The ZI‐10 can form ordered structures, modify the solvation structure, and adsorb on the interface to enhance Al deposition efficiency and decrease the energy barrier for charge transfer.

Herein, we designed an aqueous electrolyte system utilizing an amphiphilic zwitterion as an additive for in situ formed Zn–Al alloy anodes (Figure 1). In this system, Al^3+^ ions are deposited onto and alloyed with Zn foil, which addresses the passivation issue and mitigates the “tip effect” through electrostatic shielding, effectively preventing dendrites from piercing the separator and causing a short‐circuit. The amphiphilic zwitterion has the capability to self‐assemble into nanoscale aggregates, which establish an interfacially dual‐layered electric double layer (EDL), suppressing side reactions and promoting highly reversible plating/stripping. The coordination of zwitterionic species with Al^3+^ creats polarizable solvation sheath, thereby enhancing charge transfer kinetics during Al^3+^ reduction process. Therefore, this strategy enables the Zn–Al||Zn–Al symmetric cells to achieve 2500 h stable plating/stripping at 1 mA cm^−2^ and 1 mAh cm^−2^. Full cell tests with MnVO cathodes demonstrate >3300 cycles at a current density of 2 A g^−1^. Even with a high cathode mass loading of 10 mg cm^−2^, the full cells achieve nearly 100% Coulombic efficiency (CE) over 150 cycles at 2 A g^−1^ (or 20 mA cm^−2^). Moreover, Zn–Al||I_2_ full cells exhibit outstanding long‐term durability, maintaining ∼80% capacity retention even after 10000 cycles, further highlighting the potential of amphiphilic zwitterion for efficient and stable Zn–Al alloy batteries and aqueous batteries.

## 2 Results and Discussion

The amphiphilic zwitterion 3‐(decyldimethylammonio)propane sulfonate (ZI‐10), featuring a hydrophobic decyl tail and a zwitterionic headgroup, was selected as an electrolyte additive. The amphiphilicity of this compound enables its self‐assembly into ordered micelles in aqueous electrolytes. The zwitterionic portion consists of a covalently bound ammonium group (N^+^C_4_) and sulfonate group (SO_3_^−^), rendering the compound net neutral with strong polarity. Aqueous electrolytes were prepared by dissolving aluminum trifluoromethanesulfonate (Al(OTf)_3_) into water with or without ZI‐10, donated as Al(OTf)_3_/ZI‐10/H_2_O and Al(OTf)_3_/H_2_O, respectively. To assess the role of amphiphilicity, a control electrolyte was also prepared using a short‐chain zwitterion (ZI‐2), donated as Al(OTf)_3_/ZI‐2/H_2_O.

**FIGURE 2:**
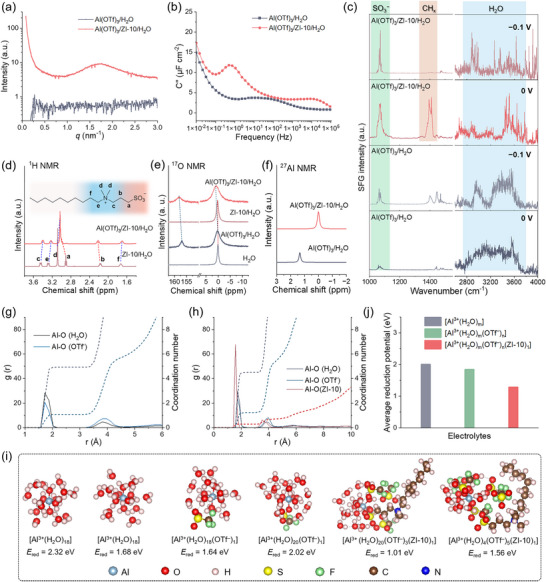
Characterization of the electrolyte structures. (a) SAXS spectra of Al(OTf)_3_/H_2_O and Al(OTf)_3_/ZI‐10/H_2_O. (b) Imaginary capacitance (*C*″) with the characteristic frequency (*f*_0_) for cells in Al(OTf)_3_/H_2_O and Al(OTf)_3_/ZI‐10/H_2_O. (c) In situ SFG spectra of Al(OTf)_3_/H_2_O and Al(OTf)_3_/ZI‐10/H_2_O. (d–f) ^1^H NMR (d), ^17^O NMR (e), and ^27^Al NMR (f) spectra of various liquids. (g,h) Radial distribution functions and corresponding coordination numbers of Zn^2+^–O (H_2_O) in Al(OTf)_3_/H_2_O (g) and Al(OTf)_3_/ZI‐10/H_2_O (h). (i) Representative optimized geometrical structures of Al^3+^(H_2_O)_m_, Al^3+^(H_2_O)_m_(OTf^–^)_n_, and Al^3+^(H_2_O)_m_(OTf^–^)_n_(ZI‐10)_1_, and thier corresponding reduction energies. (j) The average reduction energy for the Al^3+^(H_2_O)_m_, Al^3+^(H_2_O)_m_(OTf^–^)_n_, and Al^3+^(H_2_O)_m_(OTf^–^)_n_(ZI‐10)_1_.

The self‐assembly structure of ZI‐10 in the electrolyte solution was examined using small‐angle X‐ray scattering (SAXS) spectra, as shown in Figures 2a and Figure S1. In different manners from Al(OTf)_3_/H_2_O and Al(OTf)_3_/ZI‐2/H_2_O, Al(OTf)_3_/ZI‐10/H_2_O exhibits a Bragg peak at *q* = 1.73 nm^−1^, indicating the ordered nanostructures with a repeating unit length scale of ∼3.6 nm, which is nearly twice the theoretical length of the ZI‐10 molecule. The formation of ordered aggregates in Al(OTf)_3_/ZI‐10/H_2_O was attributed to the amphiphilic functional groups and proper alkyl chain length of ZI‐10 [46]. In other words, the hydrophilic zwitterionic region encounters H_2_O and Al^3+^/OTf^−^ ions, while the hydrophobic single‐tail regions aggregate together to form repeating units. Therefore, this structural ordering enables to improve the structural stability of the local EDL.

The interfacial modification at the local EDL structure was investigated through electrode capacitance measurements derived from electrochemical impedance spectroscopy (EIS). The imaginary component of the capacitance *C*″(*ω*), along with its characteristic frequency (*f*_0_), was shown in Figures 2b and Figure S2. For electrodes in Al(OTf)_3_/H_2_O and Al(OTf)_3_/ZI‐2/H_2_O, the *C*″(*ω*) spectra show single peaks appearing at *f*_0_ of 36 Hz and 13 000 Hz, respectively, consistent with typical EDL structure. Comparably, the electrode in Al(OTf)_3_/ZI‐10/H_2_O exhibits two distinct maxima in *C*″(*ω*) at *f*_0_ = 0.6 Hz and 13 000 Hz, suggesting the formation of a dual‐layered EDL structure [47]. This modified interfacial architecture is attributed to the adsorption of ZI‐10 on a first EDL like ZI‐2 and the self‐assembly effect of the long‐chain zwitterions that induce a second EDL on top of the first one.

We applied in situ sum frequency generation (SFG) spectroscopy to analyze the change in the interfacial structure of Al(OTf)_3_/H_2_O and Al(OTf)_3_/ZI‐10/H_2_O electrolytes in symmetric cells with/without electric field bias (Figure 2c). Upon an applied negative potential of −0.1 V, the signals of H_2_O and CH_x_ groups are markedly suppressed in Al(OTf)_3_/ZI‐10/H_2_O, while the SO_3_^−^ signal is narrowed. On the other hand, the signals for H_2_O are obvious in Al(OTf)_3_/H_2_O regardless of applied potential. These results indicate that the introduction of ZI‐10 alters the chemical composition of the EDL—the decrease in water content—by means of the structural ordering of amphiphilic zwitterions. Therefore, this dual‐layered EDL effectively reduces the water activity to inhibit HER and side reactions at the interface for reversible and stable Al plating/stripping, which is consistent with above results.

The solvation structures and interactions of electrolytes were investigated using spectroscopic methods and computational simulations. First, the effect of the zwitterionic character on the Al^3+^ solvation sheath was analyzed using nuclear magnetic resonance (NMR) spectroscopy. As shown in the H chemical environment of the additives in ^1^H NMR spectra (Figure 2d), the addition of Al(OTf)_3_ induces upfield shifts in the peaks representing the alkyl hydrogens near N^+^C_4_ of ZI‐10. In contrast, the peaks for the methylene hydrogens near SO_3_^−^ of ZI‐10 shift to downfields. These findings are attributed to the interactions of N^+^C_4_ with OTf^−^ and H_2_O, while the SO_3_^−^ coordinates with Al^3+^. We enlarged the H_2_O peak in ^1^H NMR spectra to observe the effect of added Al^3+^ salts on the chemical environment around the H_2_O molecules (Figure S3). Through coordination with the H_2_O, Al^3+^ of strong Lewis acidity significantly weakens the shielding effect of the nucleus, resulting in a shift toward downfields in the ^1^H chemical shift of H_2_O. The ^17^O NMR spectra further demonstrate the impact of zwitterions on solvation environments (Figure 2e). Upon the addition of ZI‐10, downfield shift caused by the deshielding effect are observed for the O chemical shift of H_2_O in Al(OTf)_3_/ZI‐10/H_2_O (0.86 ppm) with respect to that in Al(OTf)_3_/H_2_O (0.23 ppm). Furthermore, the O chemical shift of OTf^−^ in Al(OTf)_3_/ZI‐10/H_2_O (158.1 ppm) exhibits a downfield shift with respect to Al(OTf)_3_/H_2_O (156.9 ppm). These ^17^O NMR results indicate that the N^+^C_4_ of ZI‐10 binds with H_2_O and OTf^−^. In addition, the Al^3+^ chemical shift in Al(OTf)_3_/ZI‐10/H_2_O (0.01 ppm) undergoes an upfield shift compared to that in Al(OTf)_3_/H_2_O (1.34 ppm) (Figure 2f), indicating the increased shielding effect arising from the coordination Al^3+^ with ZI‐10. The localized high dielectric environment of zwitterionic species can enhance the polarizability of the Al^3+^ solvation sheath. During Al deposition, the reorganization of this solvation sheath is needed to accept electron from the electrode. The more polarizable Al^3+^ solvation sheath lowers the energy barrier for electron transfer, consequently reducing overpotential and suppressing electrolyte decomposition [48].

Density functional theory (DFT) calculations were conducted to evaluate the interaction strength between Al^3+^ and different coordinating species. The binding energy of Al^3+^–ZI‐10 (−11.18 eV) is larger than that of Al^3+^–OTf^−^ (−9.90 eV) and Al^3+^–H_2_O (−6.78 eV), indicating the strong coordination affinity of ZI‐10 toward Al^3+^ (Figure S4). Such strong interaction suggests that ZI‐10 can effectively compete with solvent molecules and anions for coordination, thereby reconstructing the initial solvation structure of Al^3+^.

To further elucidate the solvation configuration at the molecular level, molecular dynamics (MD) simulations were performed. As shown by the radial distribution function of the two electrolytes (Figure 2g,h), the *g*(*r*) profiles of Al^3+^–O (H_2_O), Al^3+^–O (OTf^−^) and Al^3+^–O (ZI‐10) with sharp peaks at around 2.0 Å are assigned to the first solvation sheath of Al^3+^ ions coordinating with H_2_O/OTf^−^/ZI‐10. In the baseline Al(OTf)_3_/H_2_O electrolyte, the coordination numbers of H_2_O and OTf^−^ around Al^3+^ are 4.93 and 1.07, respectively (Figure 2g). Upon the introduction of ZI‐10, a substantial portion of ZI‐10 participates in modifying the Al^3+^ primary solvation shell. As shown in Figure 2h, the coordination numbers of H_2_O, OTf^–^, and ZI‐10 around Al^3+^ become 4.50, 1.22, and 0.28, respectively. Based on these observations, the representative primary solvation structures in the Al(OTf)_3_/H_2_O and Al(OTf)_3_/ZI‐10/H_2_O electrolytes are [Al(H_2_O)_5_(OTf)_1_]^2+^ and [Al(H_2_O)_4_(OTf)_1_(ZI‐10)_1_]^2+^, respectively (Figure S5). Moreover, the *g*(*r*) profiles reveal that the abscissa of Al–O (ZI‐10) peak is smaller than that of Al–O (H_2_O) peak and Al–O (OTf^–^) peak, indicating a stronger coordination interaction between ZI‐10 and Al^3+^. Consequently, it is confirmed that ZI‐10 plays a significant role in reshaping the Al^3+^ solvation structure by displacing H_2_O from the primary solvation shell and increasing the polarizability of the solvation environment. The distribution of ZI‐10 modifies the local electrostatic field and thus influences the coordination configuration of Al^3+^ within the EDL region. As a result, the reconstructed solvation structures identified by MD simulations are expected to be particularly relevant to the interfacial Al species involved in the deposition process.

We hypothesize that the reduction of Al^3+^ ions, which possess an extremely high charge density, is kinetically limited by interfacial electron transfer rather than by bulk ion diffusion. The polarizable Al^3+^ solvation sheath induced by the ZI‐10 coordination is expected to lower the energy barrier associated with this charge transfer [49]. To evaluate the energetics of the first electron‐transfer step from the electrode to solvated Al^3+^ ions, quantum chemistry calculations were carried out to quantify the reduction energy from Al^3+^ clusters to Al^2+^ clusters, a descriptor for this process. Representative Al^3+^ cluster configurations were randomly extracted from MD simulations and subsequently geometry‐optimized at the quantum‐chemical level. As shown in Figure 2i,j, the reduction of [Al^3+^(H_2_O)_m_(OTf^–^)_n_(ZI‐10)_1_] clusters require less energy (1.56 eV) compared to that of [Al^3+^(H_2_O)_m_(OTf^–^)_n_] clusters (1.83 eV) and [Al^3+^(H_2_O)_m_] clusters (2.00 eV), confirming the role of ZI‐10 in facilitating electron transfer. Therefore, the modulation of the EDL structure by ZI‐10 is intrinsically coupled with the reconstruction of the Al^3+^ solvation environment and the reduction of the interfacial electron‐transfer barrier, ultimately influencing the deposition behavior of Al species.

**FIGURE 3:**
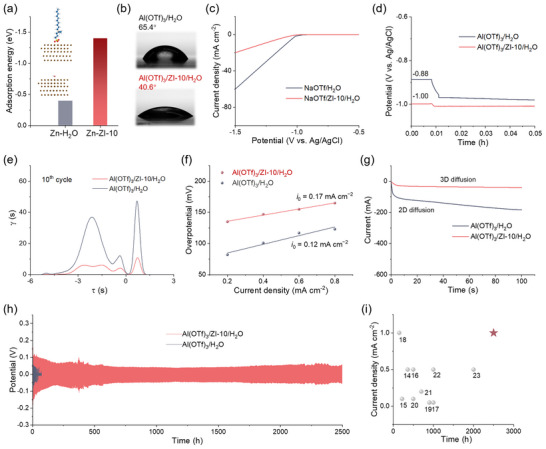
Electrolyte characteristics and their impact on the electrochemical performance of Zn–Al alloy anodes. (a) Adsorption energies of H_2_O and ZI‐10 on the Zn_(002)_ plane. (b) Contact angles of different electrolytes on Zn foil. (c) LSV curves of three‐electrode cells in different electrolytes. (d) Open‐circuit potentials and potential–time curves during the first Al deposition on the Zn substrate tested in three‐electrode cells using different electrolytes. (e) DRT spectra of a symmetric cell in different electrolytes. (f) Exchange current densities (*i*_0_) calculated from rate performance of symmetric cells in different electrolytes. (g) CA curves of symmetric cell in different electrolytes. (h) Cycling performances of symmetric cells in different electrolytes at 1 mA cm^−2^ and 1 mAh cm^−2^. (i) Comparison of symmetric cell performances with recently published Al^3+^‐assisted batteries. The numbers in the figure correspond to the references listed in the Supporting Information.

The physical and electrochemical properties of the electrolytes were investigated. To study the affinity of the electrolyte on the Zn surface, DFT calculations and contact angle measurement were conducted. As shown in Figure 3a, the adsorption energy of ZI‐10 on the Zn_(002)_ surface is –1.4 eV, much stronger than –0.4 eV of H_2_O. These values support the preferential adsorption of ZI‐10 on the Zn surface, which consequently reduces the local water content, in good agreement with the in situ SFG results. The trend of adsorption energy is reflected by contact angles related to the wettability of electrolyte (Figure 3b). The contact angles of Al(OTf)_3_/H_2_O and Al(OTf)_3_/ZI‐10/H_2_O on the Zn surface were 65.4° and 40.6°, respectively. This indicates that the electrolyte containing ZI‐10 exhibits enhanced wettability, which helps mitigate concentration polarization and promotes more uniform deposition [50].

The effect of the zwitterions on HER was verified by measuring linear sweep voltammetry (LSV) curves in a three‐electrode system, as shown in Figure 3c. To avoid interference from Al deposition, Al^3+^ ions in electrolytes were replaced by Na^+^ ions. As the cathodic sweep continues, HER gradually occurs, but ZI‐10 significantly delays the onset of this reaction. Moreover, ZI‐2 also demonstrated some ability to inhibit HER, but it is far less effective than ZI‐10 (Figure S6). Therefore, ZI‐10 effectively suppresses side reactions by modulating the solution and interfacial properties for the stable Al deposition [51].

By measuring the open‐circuit potentials in a three‐electrode cell of Zn as the working electrode, Pt as the counter electrode, and Ag/AgCl as the reference electrode, the relative Al^3+^ solvation energies in two electrolytes were evaluated (Figure 3d) [52]. The open‐circuit potentials decrease from Al(OTf)_3_/H_2_O (−0.88 V) to Al(OTf)_3_/ZI‐10/H_2_O (−1.00 V), which implies the increase in the Al^3+^ solvation energy attributed to the strong coordination of Al^3+^ with ZI‐10. This further validates a more negative Al deposition potential of Zn–Al alloy anode in Al(OTf)_3_/ZI‐10/H_2_O electrolyte than in Al(OTf)_3_/H_2_O, which allows higher cell voltages of the corresponding full cells.

The impact of the modification by ZI‐10 on the charge transfer kinetics was investigated using distribution of relaxation time (DRT) analysis to decouple the contributions from different processes. As shown in Figure 3e (Figures S7 and S8), the introduction of ZI‐10 leads to a pronounced decrease in the charge‐transfer resistance (*R*_ct_). This reduction in *R*_ct_ is attributed to the more polarizable Al^3+^ solvation environment, which reduces the energy barrier for charge transfer. Meanwhile, the diffusion‐related resistance is also significantly suppressed, suggesting improved ion transport dynamics associated with the ordered EDL structure. Further kinetic insights were gained through analysis of the exchange current density (*i*_0_), derived from the rate fitting (Figure S9). As shown in Figure 3f, the Zn–Al||Zn–Al symmetric cell employing Al(OTf)_3_/ZI‐10/H_2_O exhibits an *i*_0_ value of 0.17 mA cm^−2^, ∼1.4 times higher than that of the cell using Al(OTf)_3_/H_2_O (0.12 mA cm^−2^).

The key factors of ion transport, viscosity, ionic conductivity, and Al^3+^ transference number (*t*_Al_) were measured (Figures S10). Upon the addition of ZI‐10, the increased proportion of organic components leads to an increase in viscosity from 4.75 to 10.79 mPa s. Accordingly, the ionic conductivity decreases from 38.8 to 29.7 mS cm^−1^. The *t*_Al_ value is 0.45 for Al(OTf)_3_/ZI‐10/H_2_O (Figure S11), while that for Al(OTf)_3_/H_2_O cannot be accurately determined owing to the severe HER. The unfavorable effects of increased viscosity and reduced ionic conductivity in Al(OTf)_3_/ZI‐10/H_2_O can be compensated by the ordered interfacial structure and fast reaction kinetics. Diffusion behavior, analyzed through chronoamperometry (CA) measurements (Figure 3g), reveals that the Al(OTf)_3_/ZI‐10/H_2_O exhibits three‐dimensional (3D) diffusion, effectively suppressing dendritic growth and enabling uniform Al deposition [53]. In contrast, two‐dimensional (2D) diffusion is dominant in Al(OTf)_3_/H_2_O, which leads to unstable Al deposition and uneven alloy formation. Moreover, Al(OTf)_3_/ZI‐2/H_2_O shows a mixed 2D/3D behavior, underscoring the importance of the ordered structure on the 3D ion diffusion (Figure S12).

The electrochemical behavior of symmetric Zn–Al||Zn–Al cells was evaluated in different electrolytes. Considering the acidic nature of the Al(OTf)_3_‐based electrolyte, the dissolution of Zn to generate Zn^2+^ is unavoidable. Therefore, Al^3+^ plays a dominant role in the initial deposition process, while Zn^2+^ and Al^3+^ subsequently coexist and jointly participate in the electrochemical reactions. Under such conditions, the plating/stripping behavior should be understood as a coupled Zn–Al alloy electrode reaction, particularly in the presence of ZI‐10. (Table S1). To determine the optimal concentration of ZI‐10, the molalities were changed into 0.5, 1, and 1.5 m (mol kg^−1^) (Figure S13). Under a current density of 1 mA cm^−2^ and an areal capacity of 1 mAh cm^−2^, the plating/stripping was preserved for 1000, 2500, and 700 h, respectively, which indicates an optimal concentration of 1 m in this range. Too high concentrations lead to increased viscosity, which further reduces ionic conductivity, while too low concentrations may fail to achieve complete surface coverage and significantly modify the EDL. At the identical concentration, the plating/stripping performances of the symmetric cells were tested in Al(OTf)_3_/H_2_O, Al(OTf)_3_/ZI‐2/H_2_O, and Al(OTf)_3_/ZI‐10/H_2_O at 1 mA cm^−2^ and 1 mAh cm^−2^ (Figure 3h, Figures S14 and S15). The three electrolytes exhibit operating durations of 30, 114, and 2500 h, respectively. The enhancement effect of ZI‐2 on cell cycling stability is inferior to that of ZI‐10. This would be attributed to ZI‐2's shorter molecular structure and its lack of self‐assembly characteristics. Note that compared with the recently published research on Al^3+^‐assisted batteries (Figure 3i, Table S2), our ZI‐10 in the symmetric cells achieves the longest cycling time at quite high current density and areal capacity. These results highlight the importance of structural organization and solvation regulation by ZI‐10 on the dendrite‐free uniform Al deposition and suppressed side reaction.

The rate performance of the Zn–Al||Zn–Al symmetric cells was evaluated by increasing the current rate up to 5 mA cm^−2^ (Figure S16). In the case of the Al(OTf)_3_/H_2_O electrolyte, the cell experiences a short circuit when the current density reaches 3 mA cm^−2^. By contrast, the symmetric cells with the addition of ZI‐10 exhibit remarkable rate performances. They operated stably at a current density of 5 mA cm^−2^, demonstrating an extended duration of 150 h at the subsequent high current rate of 4 mA cm^−2^. However, under the subsequent high current density of 4 mA cm^−2^, ZI‐2 could only maintain stable plating/stripping for approximately 60 h. Therefore, ZI‐10 plays a crucial role in improving the battery's current tolerance and reaction stability under high current density.

The average Coulombic efficiency (CE) was evaluated over 200 cycles on a Cu electrode (Figure S17). Under the conditions of 1 mA cm^−2^ and 1 mAh cm^−2^, the asymmetric cell in the Al(OTf)_3_/ZI‐10/H_2_O electrolyte exhibited highly reversible and stable electrochemical cycling, achieving an average CE of 97.3%. In stark contrast, the cell in Al(OTf)_3_/H_2_O failed to sustain stable cycling after only 30 cycles, rendering the CE unquantifiable, which is attributed to severe parasitic side reactions at the interface.

**FIGURE 4:**
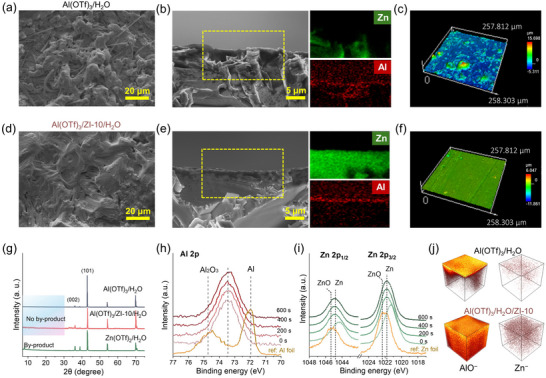
(a) Surface SEM image, (b) cross‐section SEM image and related EDX mapping, and (c) LSCM image for Zn–Al alloy anodes using Al(OTf)_3_/H_2_O. (d) Surface SEM image, (e) cross‐section SEM image and related EDX mapping, and (f) LSCM image for Zn–Al alloy anodes using Al(OTf)_3_/ZI‐10/H_2_O. (g) XRD spectra for anodes using different electrolytes. (h,i) XPS depth analysis for Zn–Al alloy anodes using Al(OTf)_3_/ZI‐10/H_2_O. (j) 3D ToF‐SIMS images of Zn–Al anodes after 20 cycles in Al(OTf)_3_/H_2_O and Al(OTf)_3_/ZI‐10/H_2_O.

To understand the correlation between the Al deposition behaviors and electrolyte structures, we examined the interface characteristics of Zn–Al alloy anodes after 20 plating/stripping cycles. Surface morphologies of the Zn–Al alloy anodes were first observed using scanning electron microscopy (SEM), as shown in Figure 4a,d. Owing to the electrostatic shielding effect of Al^3+^, the “tip effect” is eliminated. As a result, the Zn–Al alloy anodes in both Al(OTf)_3_/H_2_O and Al(OTf)_3_/ZI‐10/H_2_O exhibit significantly reduced dendrite formation compared to the Zn metal anodes cycled in typical Zn‐ion batteries (Figure S18). Nonetheless, the alloy anode in Al(OTf)_3_/ZI‐10/H_2_O exhibits a smoother and less porous surface than that in Al(OTf)_3_/H_2_O. This improvement is attributed to the ZI‐10‐modified dual‐layered EDL that homogenizes the electric field and Al^3+^ flux, thereby promoting uniform deposition [54]. By further examining the cross‐sectional SEM images (Figure 4b,e), we observed significant differences in the deposition structure. The alloy anode formed in the Al(OTf)_3_/ZI‐10/H_2_O electrolyte exhibits high uniformity at the interphase. The interphase region shows a continuous and dense alloy phase, with no visible cracks or voids between layers, indicating that the Al^3+^ migration during deposition was highly controlled. EDX mapping further confirms the uniform embedding of Al^3+^ at the interface, with a well‐distributed presence of both Al and Zn. On the other hand, the cross‐sectional images observed in the Al(OTf)_3_/H_2_O electrolyte show a large number of irregularly shaped deposition particles that are loosely and unevenly packed. This loose structure compromises the mechanical strength of the deposition layer and could also cause layer fracture or detachment during subsequent electrochemical cycling. Further evidence of the deposition assisted by ZI‐10 was obtained through laser scanning confocal microscopy (LSCM) images (Figure 4c,f). The Al(OTf)_3_/ZI‐10/H_2_O electrolyte significantly smoothens the surface of the alloy anode, resulting in a more uniform morphology compared to that obtained with the Al(OTf)_3_/H_2_O electrolyte. Such a uniform morphology suggests a more regulated Al deposition process, which can be attributed to the ZI‐10‐induced modification of the interfacial environment and the reduced electron‐transfer barrier, ultimately contributing to the formation of a stable alloy interface.

The microstructure of in situ formed Zn–Al alloy was analyzed through XRD spectra (Figure 4g). XRD peaks solely corresponding to Zn were captured, with no new peaks related to the Al element. Meanwhile, a shift in the Zn peak was observed (Figure S19). This demonstrates that during the alloying process at the electrode interface, Al is embedded into the Zn lattice, resulting in the formation of a stable amorphous alloy phase that causes lattice distortion, rather than forming distinct crystalline structures. While comparing the alloyed surface formed in Al salt‐based electrolyte to that of a typical Zn electrode cycled in Zn salt‐based electrolyte, no peaks corresponding to alkaline by‐products were observed in the 2θ = 10°–30° range for the alloyed surface. This phenomenon is typical for this Zn–Al alloy system [37]. We specifically examined the modifications induced by ZI‐10 and Al^3+^ salts on the Zn foil. The intensity ratio of the Zn (002) plane to the Zn (101) plane in the alloy cycled in Al(OTf)_3_/ZI‐10/H_2_O was found to be 0.15, higher than 0.04 of the alloy anode cycled in Al(OTf)_3_/H_2_O. In hexagonal Zn crystals, the (002) plane corresponds to the basal plane with relatively low surface energy and a densely packed atomic arrangement [55]. A higher (002)/(101) ratio, therefore indicates a stronger basal‐plane orientation, which can promote uniform surface diffusion and interfacial reactions during electrochemical processes. Consequently, such preferential orientation is conducive to forming a more compact Zn–Al alloy morphology. This observation is consistent with the homogeneous alloying results observed by EDX mapping [37].

In‐depth XPS elemental analysis was conducted to confirm the composition of the alloy phase. As illustrated by the Al 2p spectrum (Figure 4h), a signal consistently appears at a binding energy of approximately 73.4° throughout the depth profiling process. This signal lies between that of Al^0^ (around 71.9°) and Al^3+^ (around 74.7°), distinctly differing from the spectrum of the cycled Al foil used as a reference [56]. This observation provides compelling evidence for the formation of an alloy phase. For the Zn 2p spectrum (Figure 4i), the reference Zn foil displays a mixed Zn^0^ and Zn^2+^ signal [57]. In contrast, the signal from the alloy cycled in Al(OTf)_3_/ZI‐10/H_2_O electrolyte aligns with the Zn^0^ mode, signifying alloy formation and the effective suppression of side reactions.

To further validate the uniform alloy formation facilitated by ZI‐10, 3D time‐of‐flight secondary ion mass spectrometry (ToF‐SIMS) analysis was employed. The results, depicted in Figure 4j and Figure S20, demonstrate that in the alloy cycled with Al(OTf)_3_/ZI‐10/H_2_O, the AlO^−^ Al^−^ and Zn^−^ signals remained stably distributed even with increasing etching depth. Conversely, in the alloy cycled with Al(OTf)_3_/H_2_O, a noticeable inconsistency in the distribution of Al and Zn elements was observed. The more stable depth distribution observed in the ZI‐10‐containing electrolyte indicates the formation of a continuous and homogeneous interfacial alloy layer. This behavior can be attributed to the interfacial regulation induced by ZI‐10, which modifies both the EDL structure and the local solvation environment, thereby promoting more uniform nucleation and deposition of Al species at the Zn surface. Once a homogeneous nucleation process is established, the subsequent alloying between deposited Al and the Zn substrate can proceed uniformly across the interface, leading to a compact and continuous Zn–Al alloy layer. The stabilized alloy structure, as a result, underpins the formation of a robust, long‐lasting alloy anode, ultimately ensuring good cycling stability and extended lifespan for Zn–Al alloy batteries.

**FIGURE 5:**
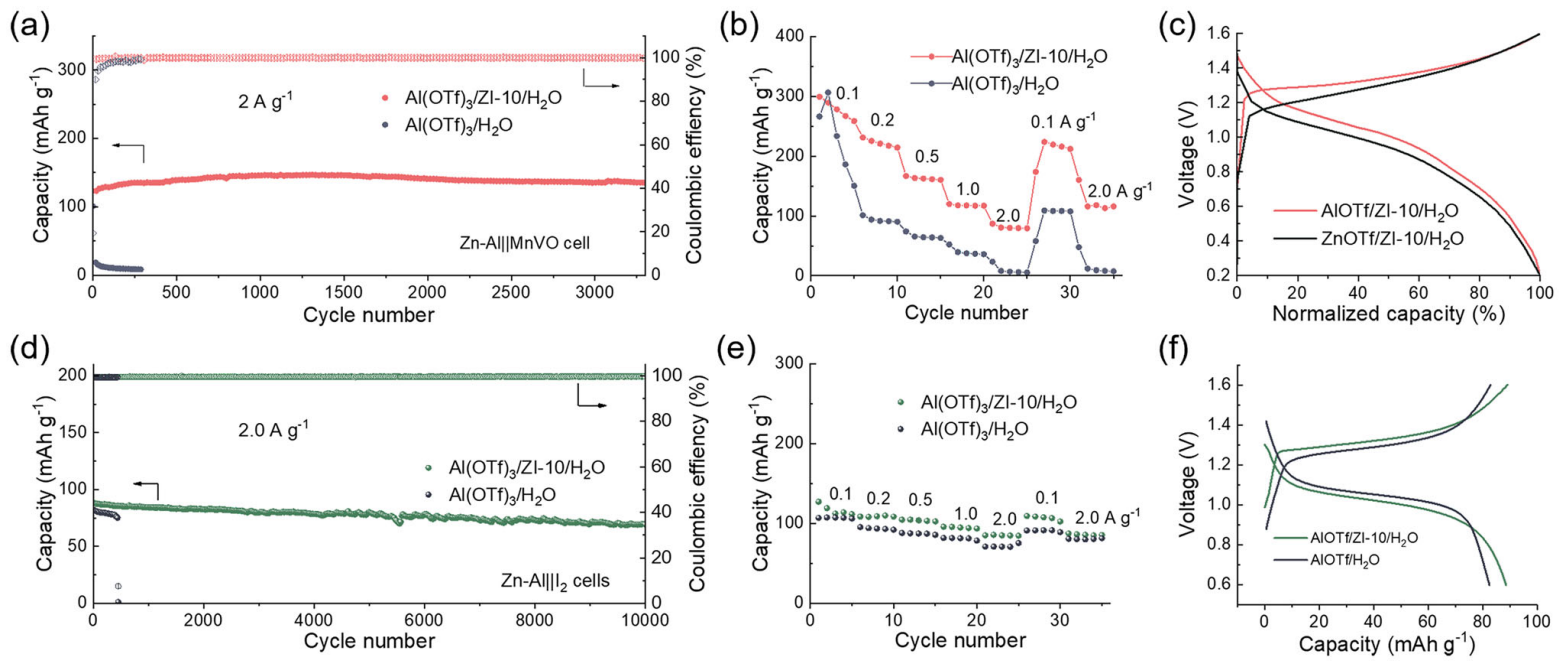
(a) Cycling performances of Zn–Al||MnVO in different electrolytes at 2 A g^−1^. (b) Rate performances of Zn–Al||MnVO in different electrolytes at 0.1, 0.2, 0.5, 1, 2, 0.1, and 2 A g^−1^. (c) Galvanostatic charge/discharge profiles of Zn–Al||MnO_2_ full cells in Al(OTf)_3_/ZI‐10/H_2_O and Zn(OTf)_2_/ZI‐10/H_2_O at 2 A g^−1^. (d) Cycling performances of Zn–Al||I_2_ in different electrolytes at 2 A g^−1^. (e) Rate performances of Zn–Al||I_2_ in different electrolytes at 0.1, 0.2, 0.5, 1, 2, 0.1, and 2 A g^−1^. (f) Galvanostatic charge/discharge profiles of Zn–Al|| I_2_ in different electrolytes at 2 A g^−1^.

To further validate the performance enhancement induced by ZI‐10 in alloy anodes, we assembled full cells for in‐depth testing. As shown in Figure 5a and Figure S21, Zn–Al||MnVO full cells with a mass loading of 4 mg cm^−2^ were tested at a current density of 2 A g^−1^ (8 mA cm^−2^). The full cells in Al(OTf)_3_/H_2_O electrolyte quickly become inactive after only a few cycles, with capacity nearly depleted. The addition of ZI‐2 improves the cycling life, but the capacity begins to fluctuate significantly after approximately 500 cycles and eventually decays rapidly. On the other hand, the full cells with ZI‐10 exhibit stable cycling for over 3300 cycles with a discharge capacity of approximately 140 mAh g^−1^ (0.56 mAh cm^−2^) and CEs of ∼100%. For the evaluation of the rate performance of the full cells, the specific capacities were measured gradually increasing the current densities from 0.1 to 2 A g^−1^ as illustrated in Figure 5b. The full cells with ZI‐10 achieve the high discharge capacity of 272 mAh g^−1^ at 0.1 A g^−1^ and the high‐rate capacity of 80 mAh g^−1^ at 2 A g^−1^. In contrast, the full cells in baseline electrolyte exhibit a rapid decline in capacity at a low current of 0.1 A g^−1^, showing only an average capacity of 225 mAh g^−1^. Furthermore, when the current density reaches 2 A g^−1^, the capacity drops to zero, indicating a failure of the electrochemical reaction at high current densities. While ZI‐2 also demonstrates some improvement in rate performance, its effectiveness is not on par with ZI‐10 (Figure S22). At the same current densities, the average discharge capacities for the ZI‐2‐used cells are 260 mAh g^−1^ at 0.1 A g^−1^ and 53 mAh g^−1^ at 2 A g^−1^, respectively. For more practical applications, the cycling performance was tested under high cathode mass loading (10 mg cm^−2^) (Figure S23). With the aid of ZI‐10, the full cell maintains close to 100% CE over more than 150 cycles at a current density of 2 A g^−1^ (20 mA cm^−2^), delivering a stable discharge capacity of 70 mAh g^−1^ (0.7 mAh cm^−2^). The successful cycling under such high mass loading highlights the robust stability and adaptability of the electrolyte environment provided by ZI‐10.

We conducted a study on the same MnVO cathode, focusing on the changes in the transporting ions in different electrolytes, particularly distinguishing the effects of Zn^2+^ and Al^3+^ on the voltage platform. As shown in the galvanostatic charge/discharge profiles in Figure 5c, the discharge plateau obtained with the Al salt electrolyte is approximately 0.07 V higher than that with the Zn salt electrolyte. The higher discharge plateau underscores the superior energy output of Zn–Al alloys and the presence of Al^3+^, highlighting their ability to provide greater energy density at the same current density [58].

Considering the unique interfacial stabilization effect of ZI‐10 on the Zn–Al anode, together with its remarkable ability to enhance cycling stability on the cathode side, we further constructed Zn–Al||I_2_ full cells to evaluate its comprehensive regulatory role under practical operating conditions. At a high current density of 2 A g^−1^, the full cell using the Al(OTf)_3_/H_2_O electrolyte could only sustain approximately 500 cycles before suffering rapid capacity decay and eventual failure (Figure 5d). This failure mode is mainly attributed to the combination of heterogeneous Al^3+^ plating/stripping, which produces mechanically unstable interphase layers, and pronounced polyiodide shuttling, both of which accelerate the degradation of the electrochemical interface. In stark contrast, after introducing ZI‐10, the full cell exhibited a remarkable enhancement in durability, achieving over 10 000 cycles at the same current density while retaining ∼80% of its capacity. This dramatic improvement highlights that ZI‐10 effectively stabilizes plating/stripping, suppresses the accumulation of parasitic reactions, and simultaneously maintains the high reversibility of iodine redox processes, enabling the entire system to operate reliably under demanding conditions. We further examined the rate performance (Figure 5e). Across all current densities (0.1 to 2 A g^−1^), the incorporation of ZI‐10 consistently enabled the full cell to deliver higher specific capacities. Further analysis of the galvanostatic charge–discharge profiles reveals an interesting phenomenon (Figure 5f). Although the addition of ZI‐10 slightly increases the electrolyte viscosity, which leads to a modest increase in the polarization, the potential during charging nevertheless reaches the voltage plateau much more rapidly. This behavior indicates that interfacial electron transfer and local desolvation of Al^3+^ are significantly accelerated. Consistent with our earlier interfacial theory analysis, this improvement originates from the ordered interfacial microenvironment and polarizable Al^3+^ solvation constructed by ZI‐10, which reduces the reorganization energy required for Al^3+^ plating, leading to faster charge‐transfer kinetics. Overall, ZI‐10 synergistically stabilizes the Zn–Al interfacial structure and optimizes charge‐transfer dynamics, thereby simultaneously enhancing the cycling life and rate capability of the full cell.

The universality of the self‐assembled ZI‐10 strategy was further validated via assembling Zn–Al||MnO_2_ full cells (Figure S24). The cell in Al(OTf)_3_/ZI‐10/H_2_O demonstrates long cycling stability, maintaining a capacity of 60 mAh g^−1^ after 800 cycles at 0.50 mA cm^−2^. In stark contrast, the cell in Al(OTf)_3_/H_2_O electrolyte experiences a rapid decline in capacity after just 50 cycles. We also conducted rate performance tests on the Zn–Al||MnO_2_ cells using Al(OTf)_3_/ZI‐10/H_2_O to further showcase their low and high current operational capabilities (Figure S25). At 0.25 mA cm^−2^, the full cells exhibit a maximum discharge capacity of 175 mAh g^−1^. At a higher current density of 1.25 mA cm^−2^, they maintained a discharge capacity of 50 mAh g^−1^. These results indicate that the incorporation of ZI‐10 significantly enhances the electrochemical performance of the Zn–Al||MnO_2_ system, providing robust support for successful operation across various cathode materials.

## 3 Conclusion

This work identifies the amphiphilic zwitterionic additive, ZI‐10, which facilitates the stable deposition of Zn–Al alloy anodes, thereby constructing high‐performance and stable Zn–Al alloy anodes. Through the analyses of SAXS, frequency‐dependent capacitance, and SFG spectroscopy, it is confirmed that ZI‐10 constructs a dual‐layered EDL through self‐assembly, effectively suppressing the occurrence of side reactions and stabilizing the electrochemical environment during plating/stripping. NMR spectroscopy and MD simulations reveal that ZI‐10, due to its localized charge variations, significantly enhances the polarizability of Al^3+^ solvation, which promotes the charge transfer kinetics during deposition. Consequently, the Zn–Al||Zn–Al symmetric cells with added ZI‐10 exhibit >2500 h of outstanding cycling stability at 1 mA cm^−2^ and 1 mAh cm^−2^, while also demonstrating stable operation at practical high current densities (4 mA cm^−2^). Additionally, the assembled Zn–Al||MnVO full cells, after over 3300 cycles at 2 A g^−1^, maintain a discharge capacity of 140 mAh g^−1^ and achieve 150 cycles of stable operation under high loading conditions (10 mg cm^−2^). In parallel, Zn–Al||I_2_ full cells exhibit excellent long‐term cycling stability (∼80% retention after 10 000 cycles), further corroborating the robustness of the proposed electrolyte design. This study offers insights into the application of smoother Zn–Al alloy anodes and provides valuable guidelines for the design and development of electrolytes for future high‐performance aqueous batteries.

## Conflicts of Interest

The authors declare no conflicts of interest.

## Supporting information




**Supporting File**: smll73425‐sup‐0001‐SuppMat.docx.

## Data Availability

The data that support the findings of this study are available from the corresponding author upon reasonable request.
