# Supplementary material for: Structural Ordering and Polarizable Al3+ Solvation Induced via Amphiphilic Zwitterion for Highly‐Reversible Zn–Al Alloy Anodes
Source: Small. 2026 Apr 17;22(32):e14994. doi: 10.1002/smll.202514994 (PMC13244408; doi:10.1002/smll.202514994)
Supplement: Supplementary file 1 — Supporting File: smll73425‐sup‐0001‐SuppMat.docx. [file SMLL-22-e14994-s001.docx]

Supporting Information

Structural Ordering and Polarizable Al^3+^ Solvation Induced via Amphiphilic Zwitterion for Highly-Reversible Zn–Al alloy anodes

Shengyang Huang, Zuyang Hu, Yilang Liu, Dong Hyun Min, Jun Su Kim, Hongdae Lee, Sida Zhang, Guanyao Wang, Zhipeng Wen, Cheng Chao Li*, Ho Seok Park* and Qingyun Dou*

**Supporting Experimental Section**

**Electrolyte preparation**

The baseline electrolyte was prepared by dissolving 2 m (molality, mol kg^−1^) aluminum trifluoromethanesulfonate (Al(OTf)_3_, Alfa Aesar, 99%) in deionized water. For cell electrolyte formulation, two different zwitterionic compounds were separately introduced into a 2 m Al(OTf)_3_ solution: 3-(decyldimethylammonio)propane sulfonate (ZI-10, Sigma-Aldrich, ≥98%) and dimethylethylammoniumpropane sulfonate (ZI-2, Sigma-Aldrich, ≥97%). The concentration of ZI-10 was optimized to 1 m for optimal performance. For Zn-Al||MnO_2_ full cells, the extra 0.2 m Mn^2+^ was added. For Zn-Al||I_2_ full cells, the extra 0.2 m NaI was added.

**Cathodes preparation**

Manganese vanadate (MnVO) was synthesized following a previously published method^[1]^. First, 60 mg of V_2_O_5_ (Sigma-Aldrich, ≥ 98%) was added to 14 mL of water. Then, 1.5 mL of H_2_O_2_ was introduced slowly, and the solution was stirred until it turned clear. Afterward, 30 mg of MnSO_4_·H_2_O was thoroughly dissolved into the mixture. The solution was then purged with nitrogen gas before being sealed. The sealed system was aged for 24 h at 80 °C, resulting in the formation of MnVO powder. For the preparation of the cathodes, a blend of 70 wt% active material, 20 wt% Ketjen Black (KB, Lion Specialty Chemicals Co., Ltd.), and 10 wt% polyvinylidene fluoride (PVDF, Sigma-Aldrich, Mw ~180,000) was mixed in 1-methyl-2-pyrrolidone (NMP, DAEJUNG). This slurry was applied onto titanium foil and dried at 80 °C overnight, with a final MnVO loading of approximately 4.0 mg cm^−2^ per cathode.

For MnO_2_, typically, 30 mL of 0.1 M KMnO_4_ solution was combined with 30 mL of 0.6 M MnSO_4_·H_2_O and stirred continuously at room temperature for 1 h. The resulting mixture was then transferred to a 100 mL Teflon-lined autoclave and heated at 140 °C for 12 h. After that, it was washed with ethanol and deionized water several times and dried at 80 ℃ overnight. The MnO_2_ cathodes were fabricated using the same procedure as the MnVO cathodes.

For Zn-Al||I_2_ full cells, the cathodes were fabricated through the following steps: a blend of 70 wt% AC (YP-80), 20 wt% carbon black (CB), and 10 wt% poly(tetrafluoroethylene) (PTFE, Sigma-Aldrich) was mixed in ethanol (DAEJUNG). This slurry was applied onto titanium foil and dried at 80 °C overnight, with an AC loading of approximately 5.0 mg cm^−2^ per cathode.

**Cell fabrication**

The alloy electrodes for Zn-Al||Zn-Al symmetric cells were in-situ generated. Zn foil (Alfa Aesar, 100 µm thick) was punched into 15 mm diameter disks to serve as electrodes. Glass fiber (Whatman, GF/A) was used as the separator, with 90 µL of electrolyte added. The assembly of asymmetric and full cells followed a similar procedure, with the only difference being the replacement of the cathodic Zn with a 15 mm diameter Cu disk or full cell cathode. All cells were assembled in CR2032 coin cell cases.

**Material Characterizations**

The Fourier-transform infrared (FT-IR) spectra were recorded utilizing JASCO FT-IR 4700 instruments equipped with attenuated total reflection (ATR) features. For Raman spectroscopy, measurements were conducted using a confocal Raman spectrometer (NT-MDT) with an operational wavelength of 532 nm. For nuclear magnetic resonance (NMR) analysis, samples were prepared by dissolving the electrolytes in deuterium oxide (D_2_O), and the ^1^H NMR spectra were captured using a 700 MHz spectrometer (Bruker AVANCE III 700). Small-angle X-ray scattering (SAXS) data were collected using a Mar165 charge-coupled device (CCD) detector with a pixel resolution of 2048 × 2048 and a pixel size of 79 µm. The distance between the sample and the detector was maintained at 1600 mm, with an active measurement area of Φ165 mm. Sum-frequency generation (SFG) experiments were executed employing an Ekspla SFG system featuring a mode-locked Nd laser set at 532 nm. To evaluate ionic conductivity, real-time measurements were taken using a SevenMulti conductivity meter (Mettler-Toledo). A SmartDrop_Plus contact angle goniometer (FEMTOBIOMED) was utilized for measuring contact angles. The morphological characteristics of the alloy anodes were analyzed through scanning electron microscopy (SEM, JSM-7000F, JEOL), while three-dimensional imaging and reconstruction of the cycled alloy anodes were performed with an Olympus 3D laser confocal scanning microscope (OLS5100). X-ray diffraction (XRD) patterns were obtained using a PANalytical diffractometer with Cu Kα radiation (λ = 0.154 nm). The electrodes' surface composition was characterized via X-ray photoelectron spectroscopy (XPS, ESCALAB 250, Thermo-Scientific), where depth profiling was carried out using argon ion sputtering at different time intervals (0, 200, 400, and 600 s). The binding energies measured in the XPS spectra were referenced against the adventitious C 1s peak positioned at 284.6 eV. Time-of-flight secondary ion mass spectrometry (ToF-SIMS) analyses were performed using a TOF-SIMS-5 (ION-TOF), applying a 1 keV O_2_ beam for sputtering across a 150 × 150 µm^2^ area, while the analytical focus was restricted to a 40 × 40 µm^2^ region utilizing a pulsed 25 keV Bi⁺ primary beam.

**Electrochemical Measurement**

The electrochemical performance of various batteries was evaluated using the WonATech WBCS3000L battery testing system. A comprehensive analysis of the electrochemical properties of the batteries was conducted with the BioLogic VMP3 multi-channel potentiostat. The open circuit potential measurements were performed using a three-electrode configuration, with Ag/AgCl serving as the reference electrode.

For the linear sweep voltammetry (LSV) tests, stainless steel (SS) was employed as both the working and counter electrodes, while Ag/AgCl was utilized as the reference electrode. The electrochemical impedance spectroscopy (EIS) measurements of the charge transfer resistance (R_ct_) were conducted on symmetric Zn-Al||Zn-Al cells, spanning a frequency range from 100 kHz to 10 Hz. For impedance results, imaginary part of the capacitance (*C*ʺ) was calculated from the EIS data obtained during the symmetric SS||SS cell testing using the following equation^[2]^:

$$\text{C″}\text{=}\frac{\text{Z}\text{ʹ}}{\text{2π}\text{f}{\text{ |}\text{Z}\text{|}}^{\text{2}}} \text{(1)}$$

where the *f* is the frequency, the *Zʹ* is the real part of the electrochemical impedance *Z*, the *Z*ʺ is the imaginary part of *Z*, defined as |*Z*|^2^= *Zʹ*^2^+ *Z*ʺ^2^.

The exchange current density (*i*_0_) was extracted from the rate fitting according to the following equation:

$$\text{i}\text{ }\text{≈}\text{ }\text{i}\text{0}\text{ }\frac{\text{3}\text{F}}{\text{RT}} \text{η}\text{ }\text{(2)}$$

Chronoamperometry (CA) curves were obtained in symmetric Zn-Al||Zn-Al cells at a bias of 150 mV. The Al^3+^ transference number was determined using symmetric Zn-Al||Zn-Al cells, with measurements taken at a bias of 10 mV.

**Calculation Methods**

Gaussian (G09) was used for density functional theory (DFT), and the Becke’s three-parameter hybrid method was employed with the Lee-Yang-Parr correlation functional (B3LYP)^[3]^. Structural optimizations were carried out at the 6-311++G(d,p) level^[4]^, and single point energy calculations were performed using the def2-TZVP basis set^[5]^. Throughout the entire calculation process, we employed Grimme dispersion correction with Becke Johnson damping DFT-D3 (BJ)^[6]^. Consider solvation effects using the SMD universal solvation model^[7]^. Frequency analysis was conducted to confirm that all optimized ion solvent complexes correspond to the true minimum values on the potential energy surface. Perform electrostatic potential (ESP) analysis using Multiwfn^[8]^ and visualize it using VMD^[9]^ software. Calculate the binding energy (*E*_b_) between molecule/ion A and molecule/ion B according to Eq. 3:

$\text{E}_{\text{b}}\text{ }\text{= }\text{E}_{\text{complex}}-\text{E}_{\text{A}}-\text{E}_{\text{B}}$ (3)

Using the projector augmented wave (PAW) method as implemented in the Vienna Ab Initio Simulation Package (VASP) for adsorption energy calculations^[9]^. The generalized gradient approximation (GGA) using Perdew Burke Ernzerhof (PBE) functionals is used to describe the exchange correlation energy^[10]^. A cut-off energy of 500 eV for plane waves and a 3 × 3 × 1 k grid at the center of gamma were used in all calculations. Perform structural optimization until the residual energy on all atoms is less than 0.03 eV Å⁻^1^, and the total energy convergence criterion is set to 1 × 10⁻^5^. Use Eq. 4 to calculate the adsorption energy (*E*a) between the Zn plate and the adsorbate:

$\text{E}_{\text{a}}\text{= }\text{E}_{\text{system}}-\text{E}_{\text{slab}}-\text{E}_{\text{Adsorbate}}$ (4)

For quantum chemistry calculations, the reduction energy was calculated by the energy difference between the solvated Al^3+^ clusters and the solvated Al^2+^ clusters. The reorganization energy was calculated based on the Al^3+^ and Al^2+^ states. All the calculations were performed by PySCF (gpu4pyscf, V1.5) with the M05-2X functionals. The def2svp and def2tzvp basises were used for the geometry optimization and single point calculations, respectively. Solvation with H_2_O were considered by the PCM model in all calculations.

Using the Large-scale Atomic/Molecular Massively Parallel Simulator (LAMMPS) for Molecular dynamics (MD) were based on liquid simulation optimized potential full atom (OPLS-AA) force field^[11]^. The force field parameters of organic solvents were generated using a LigParGen network server, while the constrained electrostatic potential (RESP) atomic charge was obtained from electrostatic potential (ESP) fitting using Multiwfn^[8]^. The initial atomic configuration was constructed using the Packmol package^[12]^, and all final model frameworks and solvation structures were visualized using VMD. Periodic boundary conditions are applied in all three-dimensional spaces. The cutoff distance of the particle particle particle mesh (PPPM) method for van der Waals interaction and long-range Coulomb interaction is set to 10 Å. The time step is set to 1fs. All electrolyte models are first equilibrated in the NVT ensemble using a Nosé - Hover thermostat at 298 K for 5 ns^[13]^. Subsequently, a 20 ns NPT ensemble simulation was conducted under Nosé-Hover thermostat and regulator to fully balance the system at 298 K and 1 atm. Finally, a 15 ns production run was conducted under NPT conditions of 298 K and 1 atm, during which data on radial distribution function (RDF) and solvent shell analysis were collected.


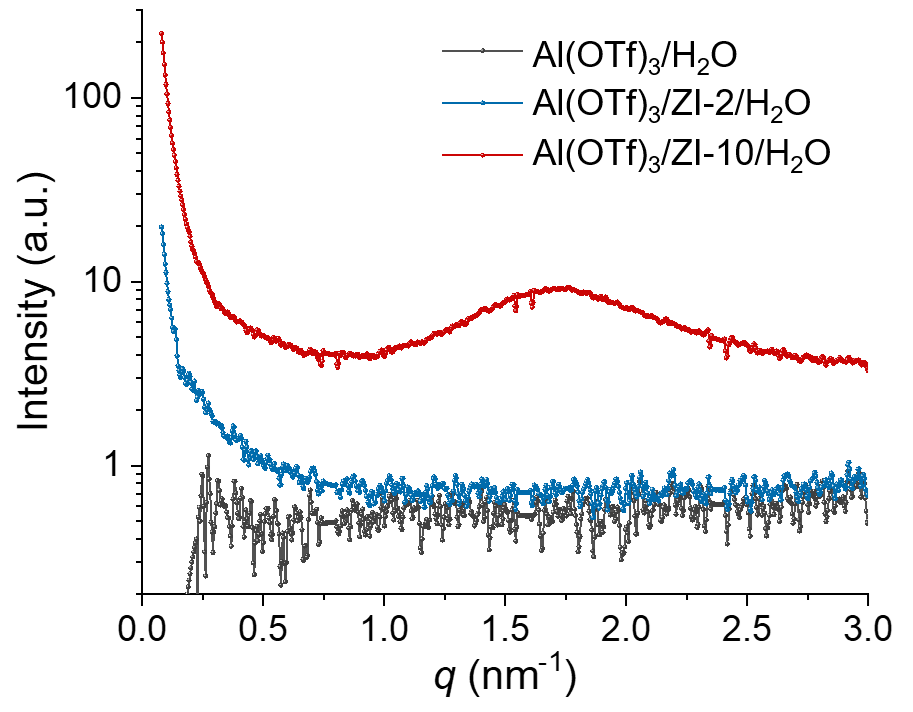


**Figure S1**. Synchronous SAXS spectra of different electrolytes.


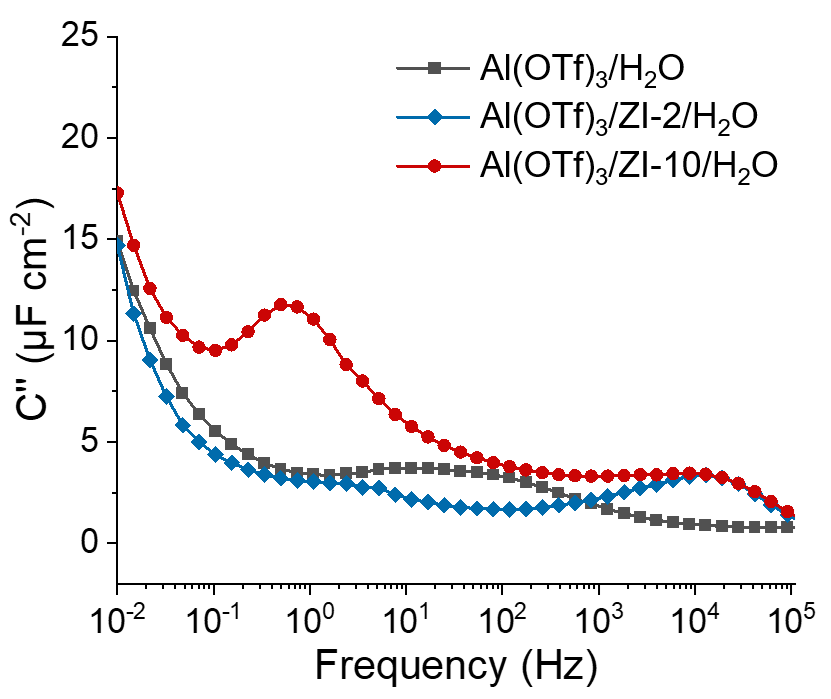


**Figure S2**. Characteristic frequency (*f*_0_) spectra of different electrolytes.

**Figure S3**. The water peaks in ^1^H NMR spectra of different electrolytes.


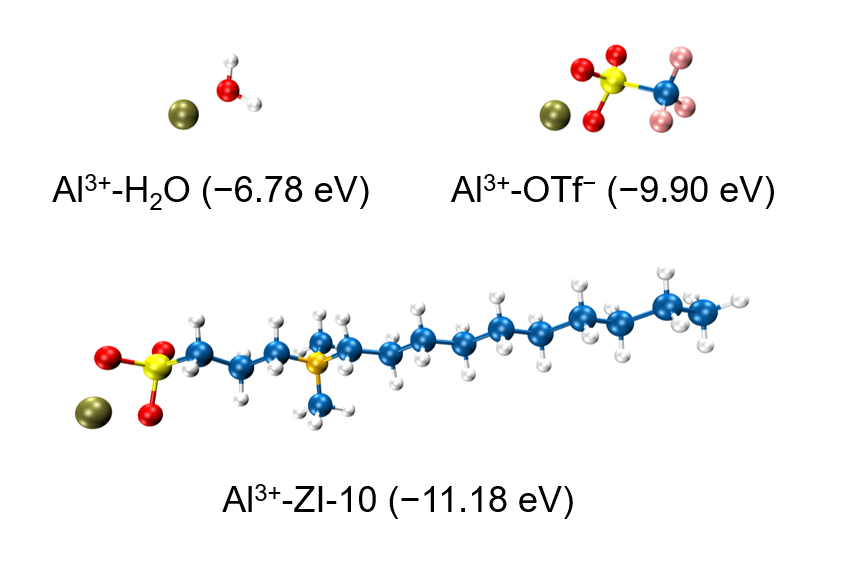


**Figure S4**. Binding energies of Al^3+^ and different species.


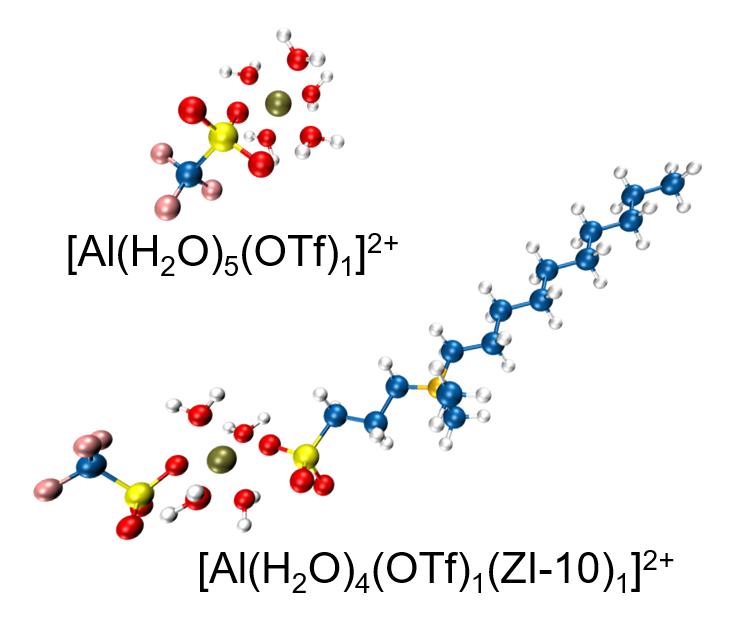


**Figure S5.** Geometric structure of [Al(H_2_O)_5_(OTf)]^2+^ and [Al(H_2_O)_4_(OTf)_1_(ZI-10)_1_]^2+^.


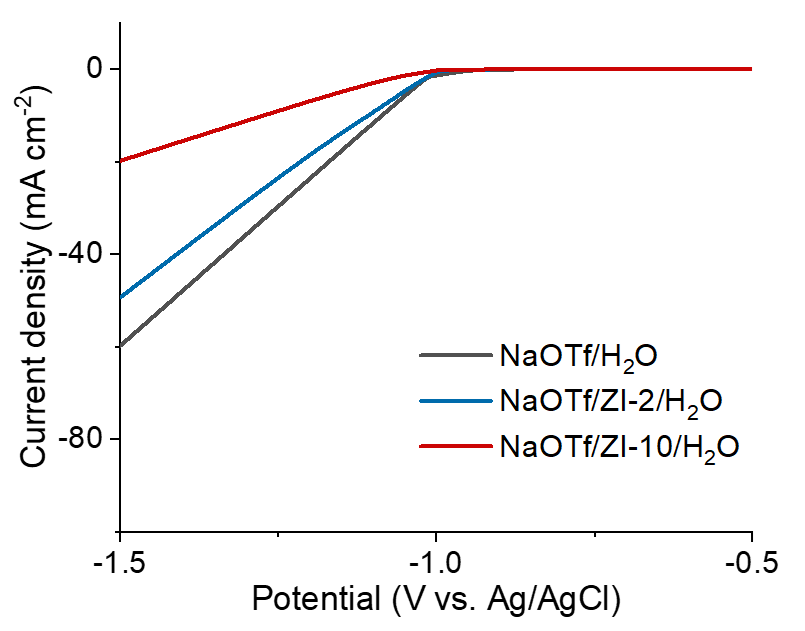


**Figure S6**. LSV curves (HER) of different electrolytes.


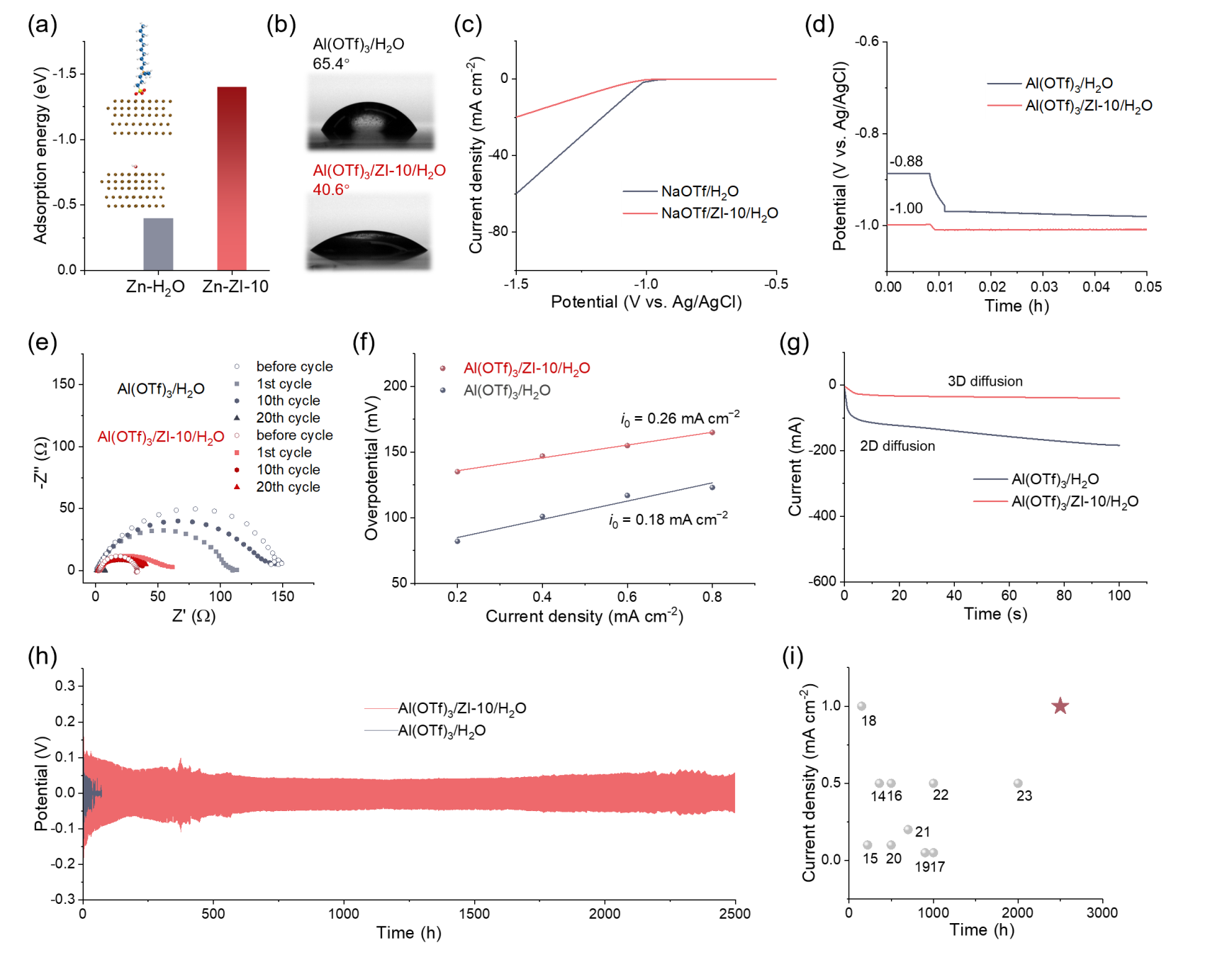


**Figure S7.** EIS results of two electrolytes before/after cycling.


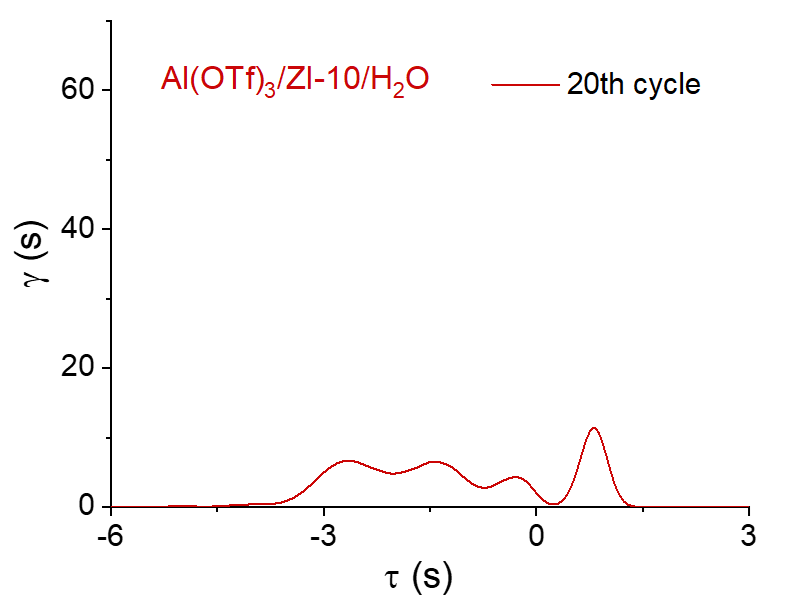


**Figure S8.** DRT result after 20^th^ cycle.

**Figure S9**. Rate perofrmance of Zn-Al||Zn-Al symmetric cell using different electrolytes cycled under various current densities and a capacity of 0.2 mAh cm^−2^.


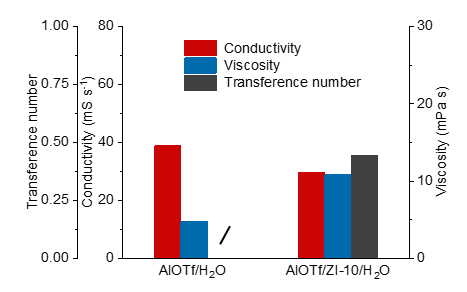


**Figure S10**. Ionic conductivity, viscosity, and transference number of different electrolytes


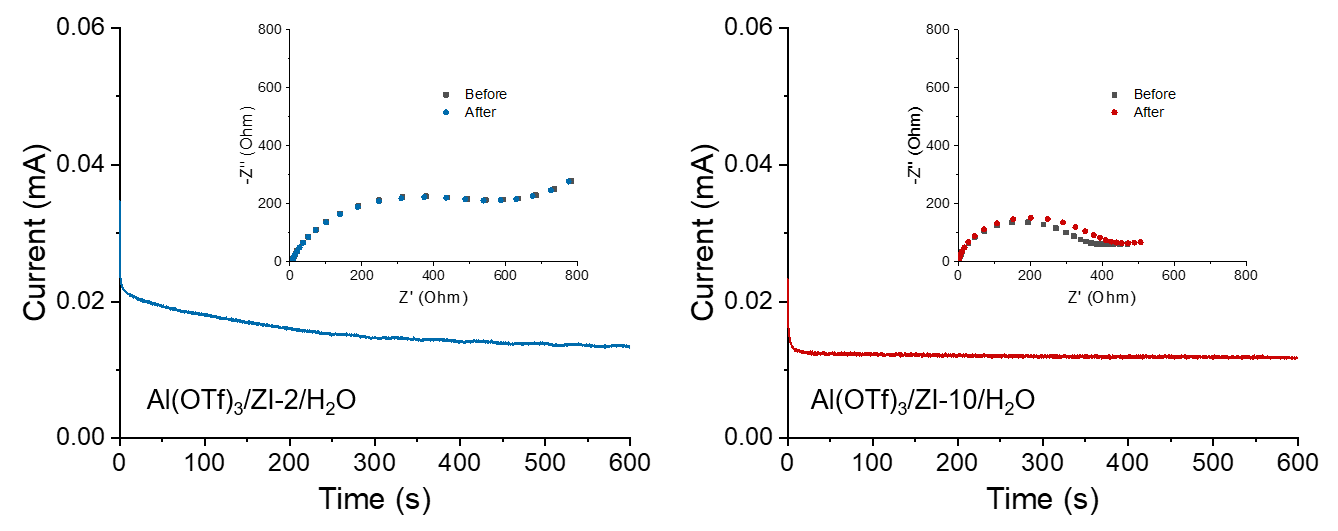


**Figure S11.** Transference number of different electrolytes.


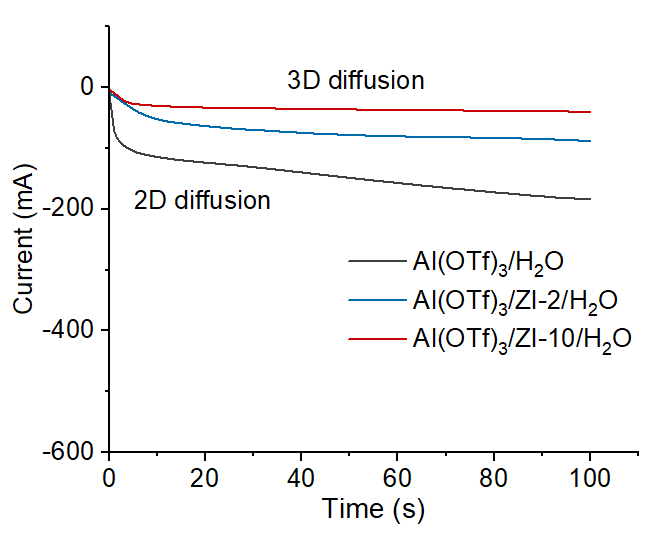


**Figure S12**. CA curves of Zn-Al||Zn-Al symmetric cells in different electrolytes.

**Figure S13**. Cycling performances of Zn-Al||Zn-Al symmetric cells in Al(OTf)_3_/H_2_O with different ZI-10 concentrations at 1 mA cm^−2^ for 1 mAh cm^−2^.


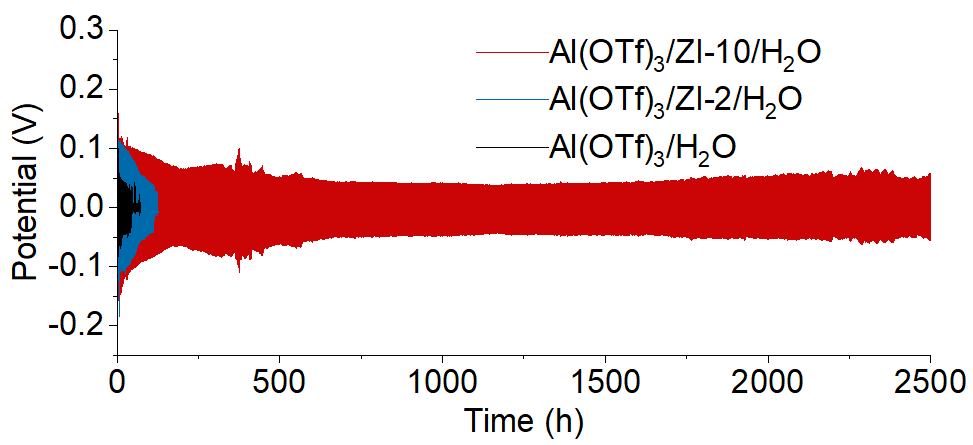


**Figure S14**. Cycling performances of Zn-Al||Zn-Al symmetric cells in different electrolytes at 1 mA cm^−2^ for 1 mAh cm^−2^.


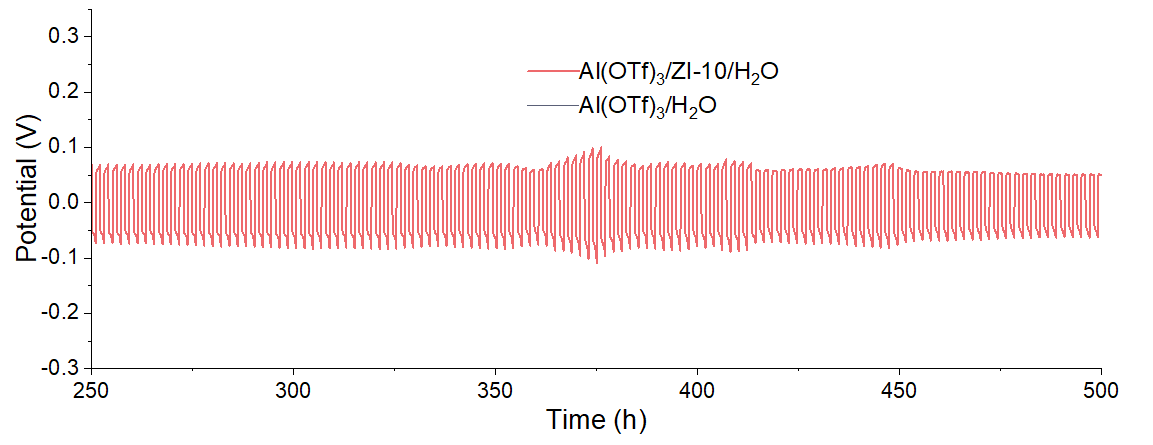


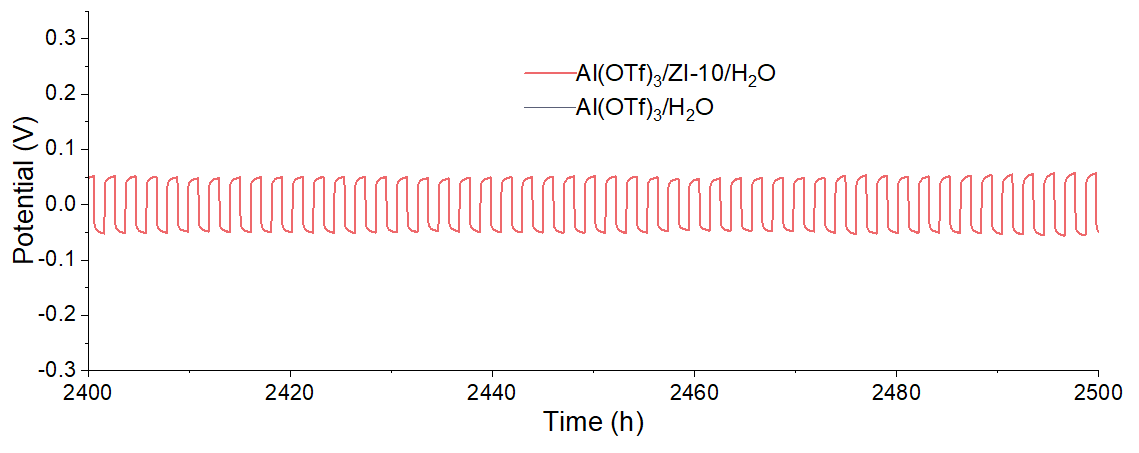


**Figure S15.** Enlarged figures of Figure 3h.


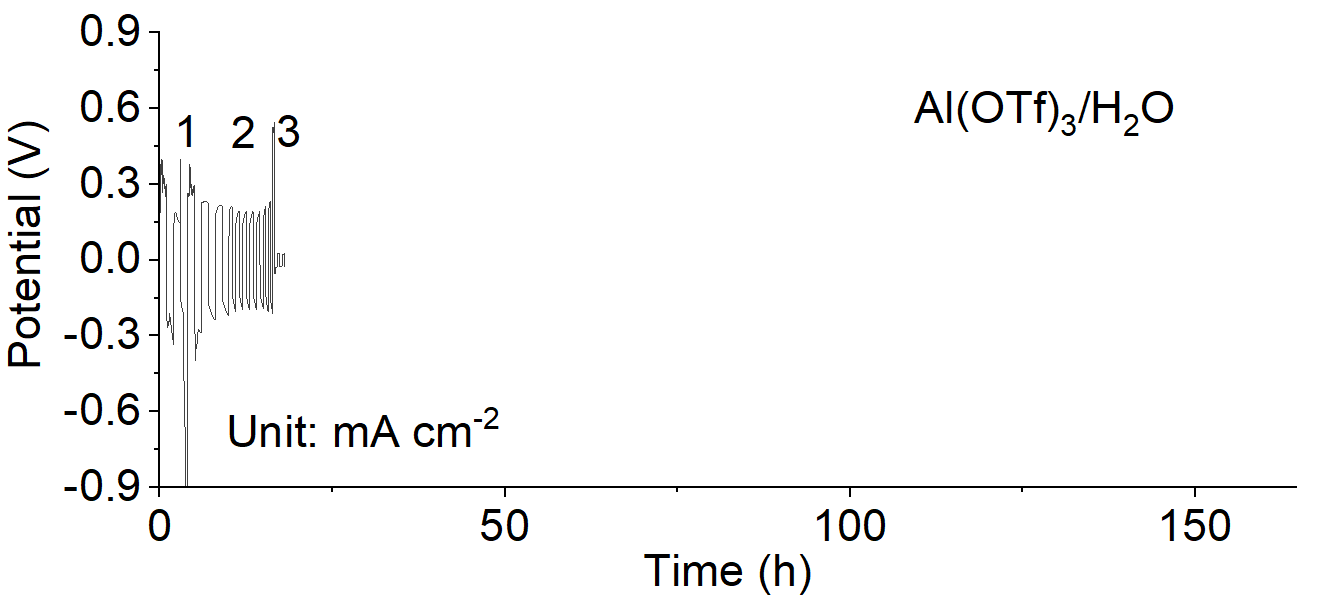


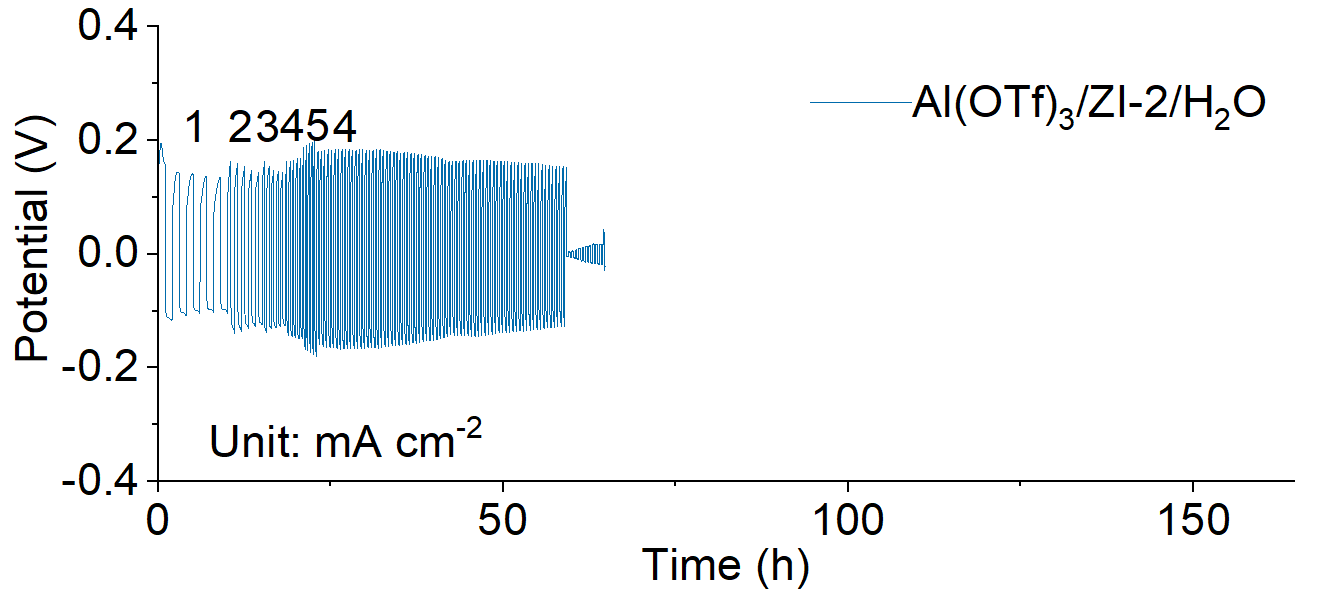


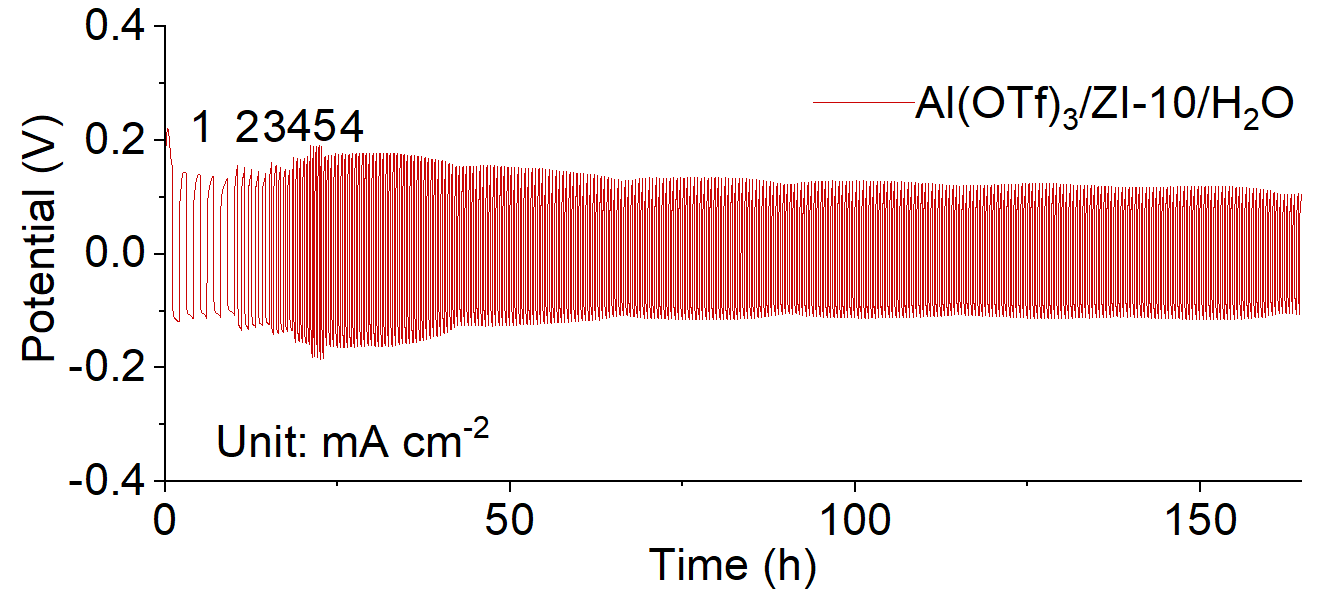


**Figure S16**. Rate performances of Zn-Al||Zn-Al symmetric cells cycled under various current densities and a capacity of 1.0 mAh cm^−2^.


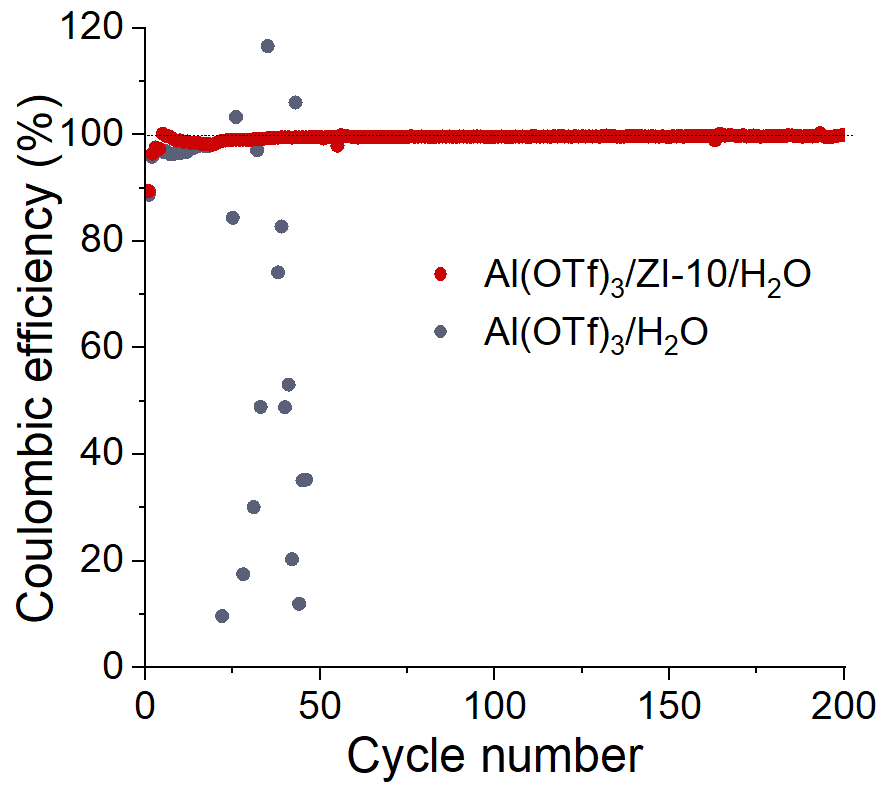


**Figure S17.** Asymmetric cells using different electrolytes at 1 mA cm^−2^ and 1 mAh cm^−2^.


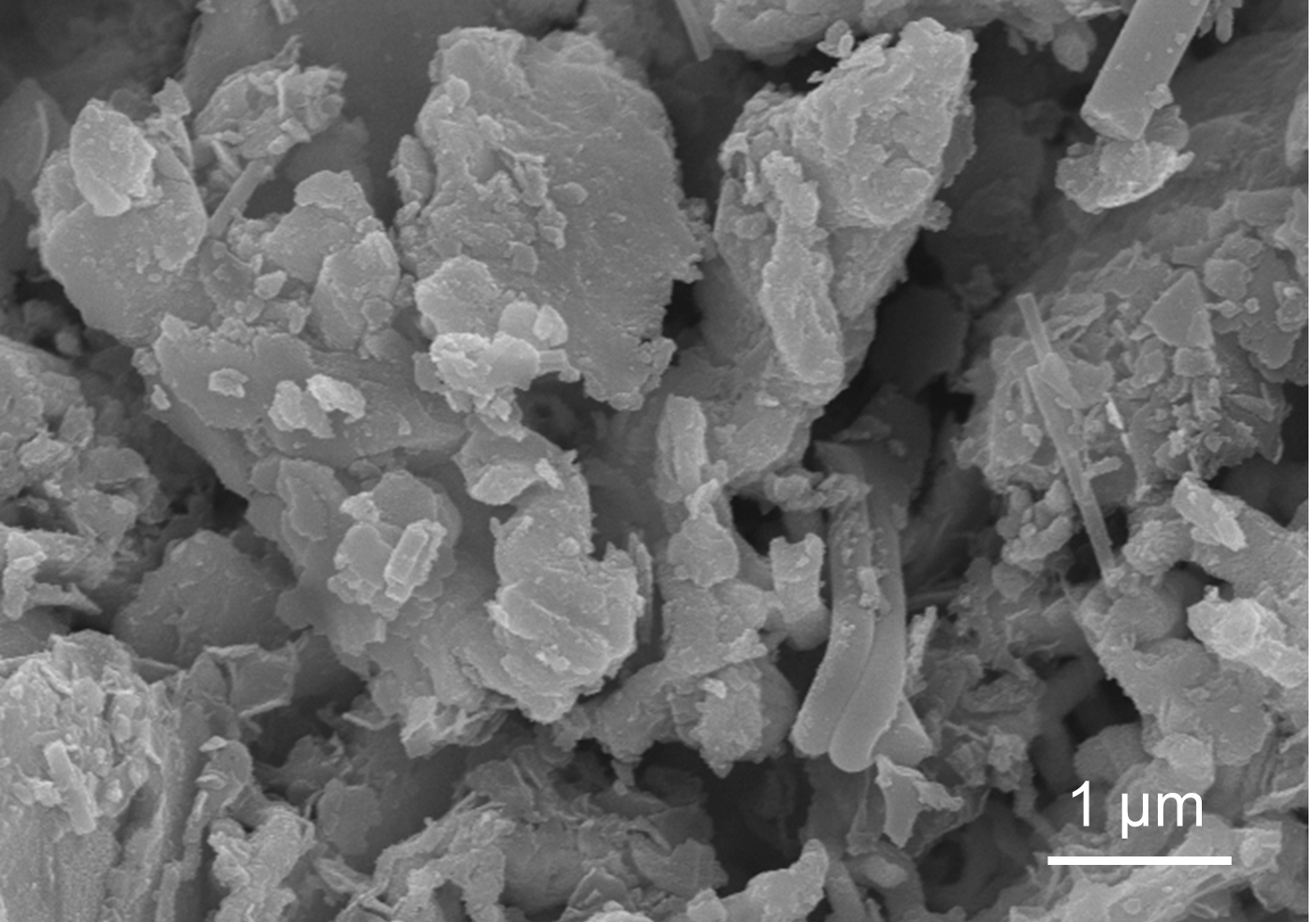


**Figure S18**. SEM image of Zn after cycling for 20 cycles in Zn(OTf)_2_/H_2_O.


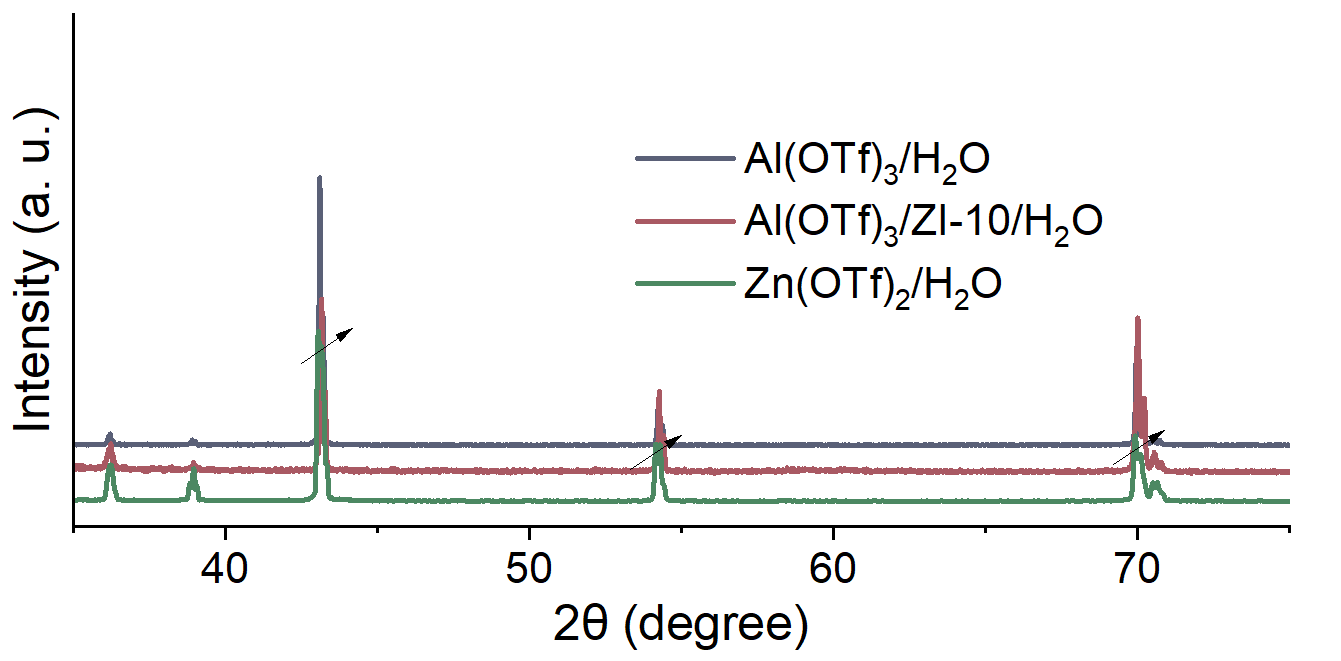


**Figure S19**. Enlarged XRD spectra for anodes using different electrolytes.


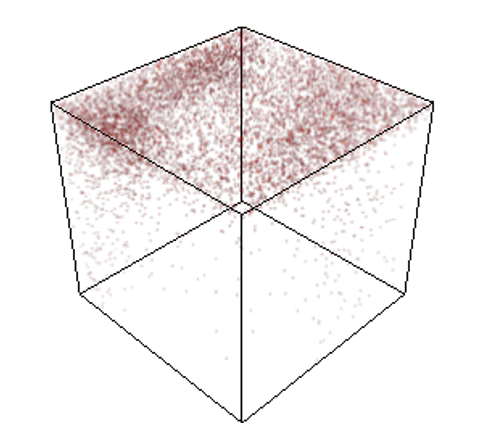

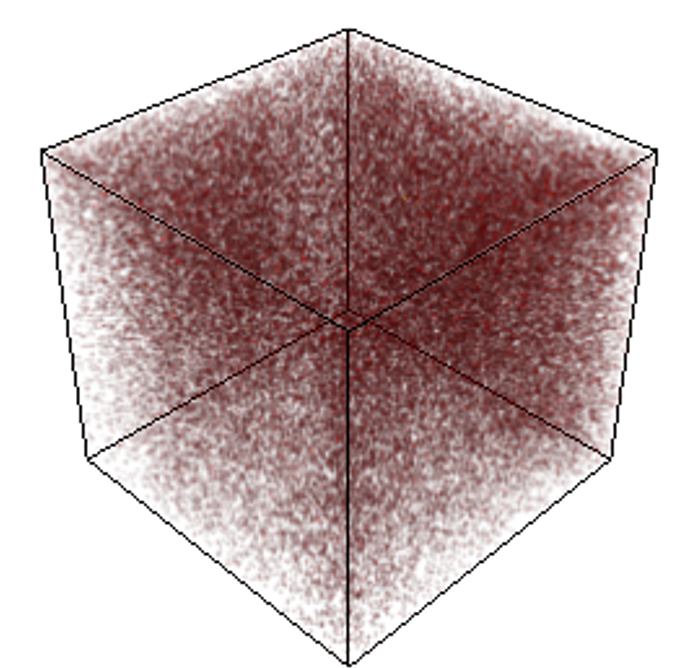


**Figure S20**. 3D TOF-SIMS image (Al^−^) of Zn-Al anodes after 20 cycles in Al(OTf)_3_/H_2_O (Left) and Al(OTf)_3_/ZI-10/H_2_O (Right).


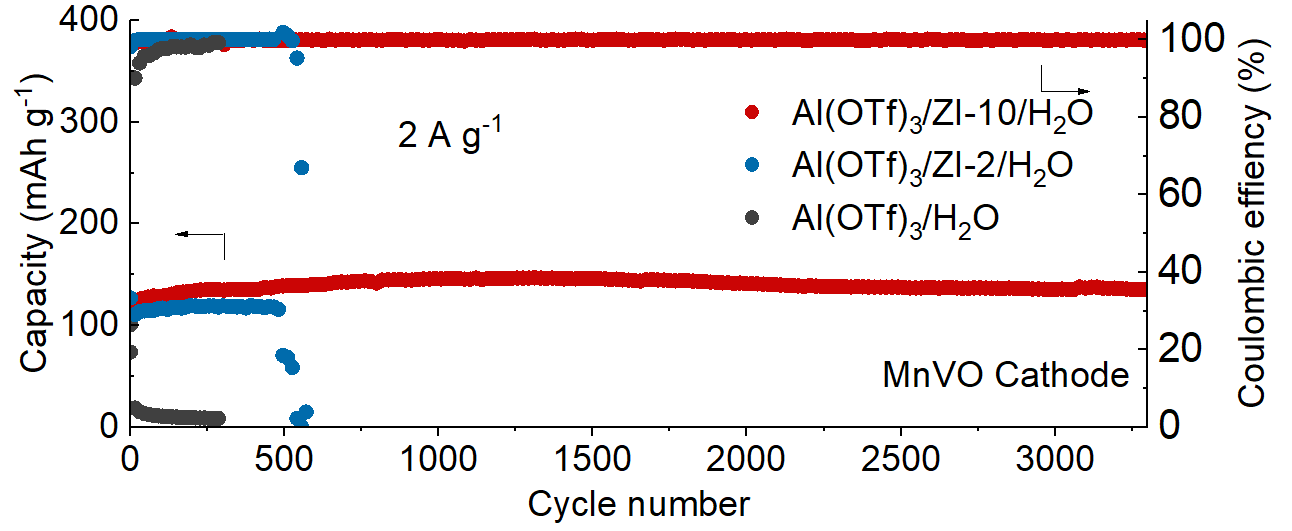


**Figure S21**. Cycling stability of Zn-Al||MnVO full cells using different electrolytes.


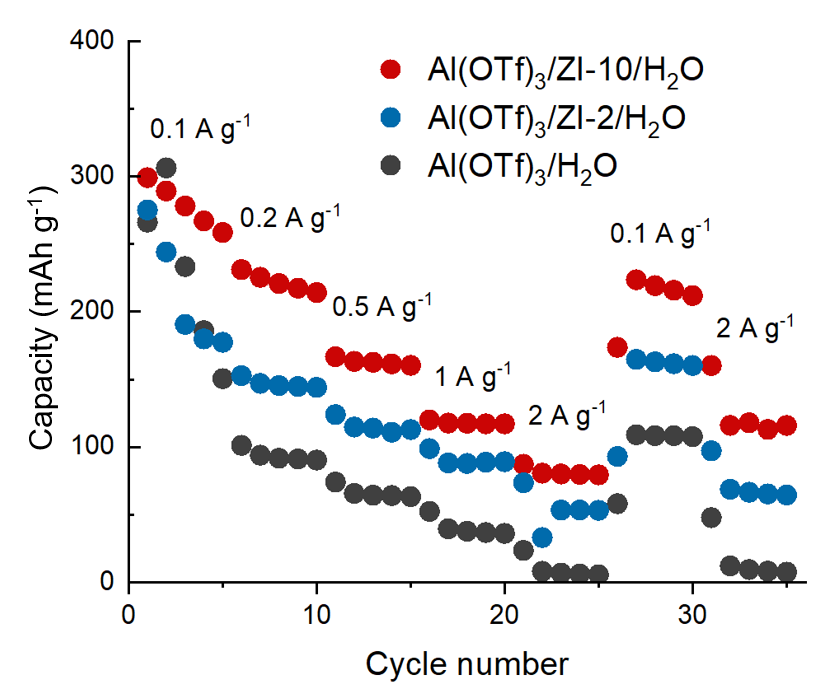


**Figure S22**. Rate performances of Zn-Al||MnVO full cells using different electrolytes.

**Figure S23**. Cycling performances of Zn-Al||MnVO in Al(OTf)_3_/ZI-10/H_2_O at 2 A g^−1^ (20 mA cm^−2^) with a cathode mass loading of 10 mg cm^−2^.


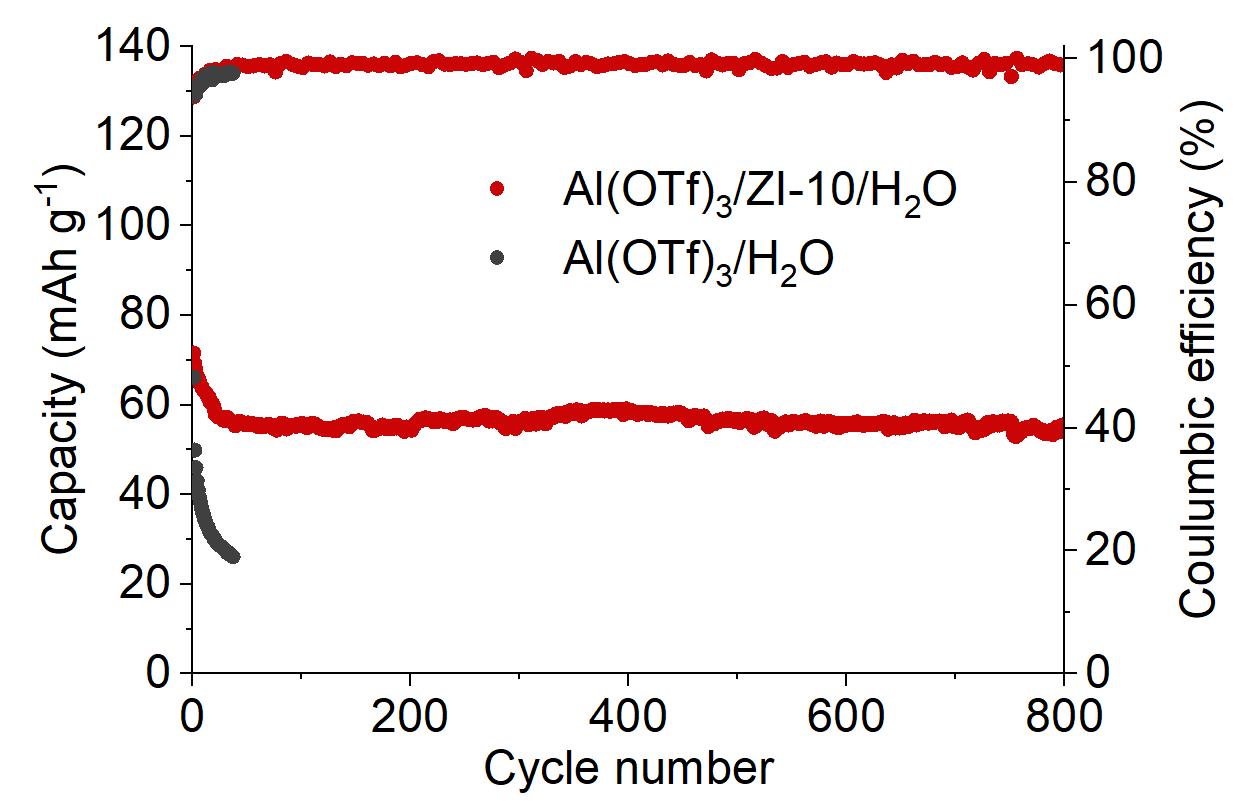


**Figure S24**. Cycling performances of Zn-Al||MnO_2_ full cells in different electrolytes at 0.50 mA cm^-2^.


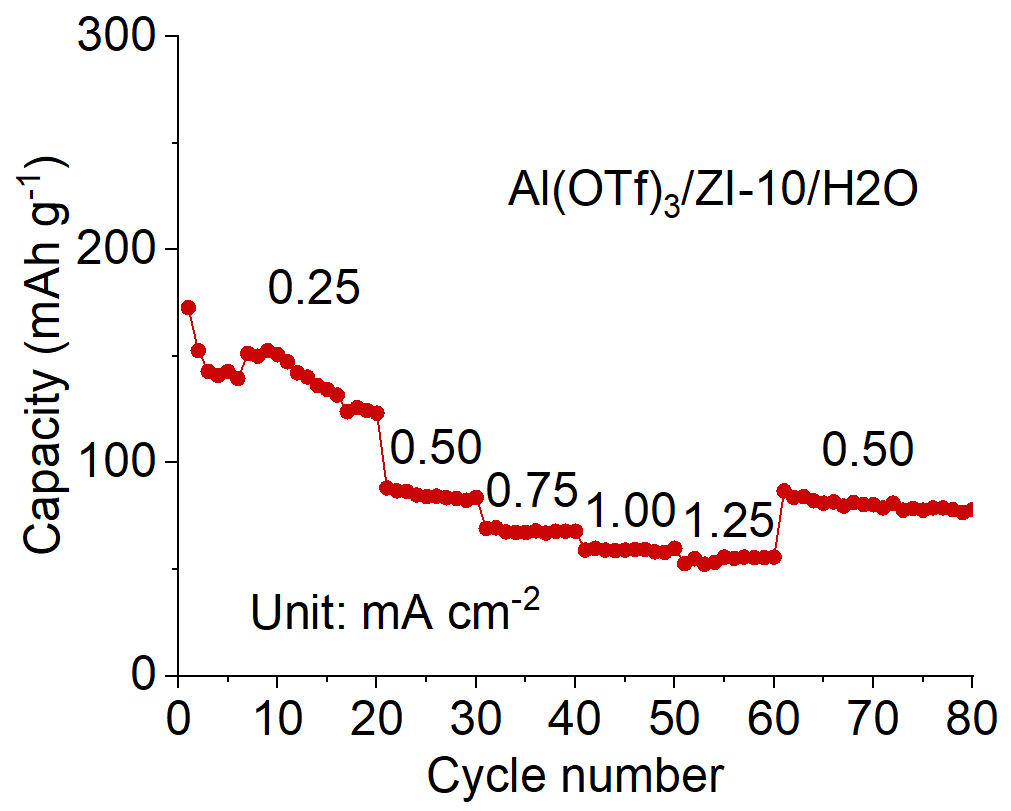


**Figure S25**. Rate performances of Zn-Al||MnO_2_ full cells in Al(OTf)_3_/ZI-10/H_2_O.

**Table S1.** ICP-OES results.

| **Electrolyte** | **Zn (mg/L)** | **Al (mg/L)** |
| --- | --- | --- |
| Al(OTf)_3_/ZI-10/H_2_O | / | 2.163 |
| Al(OTf)_3_/H_2_O after 10 cycles | 4.737 | 1.807 |
| Al(OTf)_3_/ZI-10/H_2_O after 10 cycles | 3.860 | 0.467 |

**Table S2.** Comparison of cycling performances for symmetric cells with recently reported AIBs.

| **Strategy**  **Type** | **Current density** | **Capacity** | **Time** | **Ref.** |
| --- | --- | --- | --- | --- |
| PV@ZC  Anode | 0.5 mA cm^-2^ | 0.125 mAh cm^-2^ | ~360 h | ^[14]^ |
| Acetamide  Electrolyte | 0.1 mA cm^-2^ | 0.1 mAh cm^-2^ | ~220 h | ^[15]^ |
| Sn SAC@Al | 0.5 mA cm^-2^ | 0.5 mAh cm^-2^ | 500 h | ^[16]^ |
| HEE30  Electrolyte | 0.05 mA cm^-2^ | 0.05 mAh cm^-2^ | 1000 h | ^[17]^ |
| PEG hydrogel  Electrolyte | 1 mA cm^-2^ | 5 mAh cm^-2^ | 150 h | ^[18]^ |
| Sn@Al  Anode | 0.05 mA cm^-2^ | 0.1 mAh cm^-2^ | 900 h | ^[19]^ |
| BMEA  Electrolyte | 0.1 mA cm^-2^ | 0.1 mAh cm^-2^ | 500 h | ^[20]^ |
| HE  Electrolyte | 0.2 mA cm^-2^ | 0.2 mAh cm^-2^ | 700 h | ^[21]^ |
| AU15  Electrolyte | 0.5 mA cm^-2^ | 0.125 mAh cm^-2^ | ~1000 h | ^[22]^ |
| E-Al_82_Cu_18_  Anode | 0.5 mA cm^-2^ | 0.5 mAh cm^-2^ | 2000 h | ^[23]^ |
| **ZI-10**  **Zn-Al** | 1 mA cm^-2^ | 1 mAh cm^-2^ | 2500 h | **This work** |

References

[1] S. Huang, P. Zhang, J. Lu, J. S. Kim, D. H. Min, J. S. Byun, M. J. Kim, H. Fu, P. X. Xiong, P. J. Yoo, W. W. Li, X. Yu, X. Qin, H. S. Park, *Energ Environ Sci* **2024**.

[2] J. L. Ye, Y. C. Wu, K. Xu, K. Ni, N. Shu, P. L. Taberna, Y. W. Zhu, P. Simon, *J Am Chem Soc* **2019**, 141, 16559.

[3] a)Frisch M J, Trucks G W, Schlegel H B, et al. *Gaussian revision E. 01*, Gaussian Inc, 2009. b)P. J. Stephens, F. J. Devlin, C. F. Chabalowski, and M. J. Frisch, *J. Phys. Chem.*, 1994, **98**, 11623-11627. c)A. D. Becke, *J. Chem. Phys.*, 1993*,* **98**, 5648-5652.

[4] R. Krishnan, J. S. Binkley, R. Seeger, and J. A. Pople, *J. Chem. Phys.*, 1980*,* **72**, 650-654.

[5] a)A. Schaefer, H. Horn, and R. Ahlrichs, *J. Chem. Phys.*, 1992, **97**, 2571-2577. b)A. Schaefer, C. Huber, and R. Ahlrichs, *J. Chem. Phys.*, 1994, **100**, 5829-5835.

[6] a)S. Grimme, J. Antony, S. Ehrlich, and H. Krieg, *J. Chem. Phys.*, 2010, **132**, 154104. b)S. Grimme, S. Ehrlich, and L. Goerigk, *J. Comput. Chem*., 2011, **32**, 1456-1465.

[7] B. P. Pritchard, D. Altarawy, B. Didier, T. D. Gibson, and T. L. Windus, *J. Chem. Inf. Model.*, 2019, **59**, 4814-4820.

[8] A. V. Marenich, C. J. Cramer, and D. G. Truhlar, *Phys. Chem. B*, 2009, **113**, 6378-6396.

[9] a)T. Lu, and F. Chen, *J. Comput. Chem*., 2012, **33**, 580-592. b)T. Lu, *J. Chem. Phys.*, 2024, **161**, 082503.

[10] a). Kresse, and J. Furthmüller, *Phys. Rev. B,* 1996, **54**, 11169-11186. b)J. P. Perdew, K. Burke, and M. Ernzerhof, *Phys. Rev. Lett*., 1996, **77**, 3865-3868.

[11] a)P. E. Blöchl, *Phys. Rev. B*, 1994, **50**, 17953-17979. b)S. Plimpton, *J. Comput. Chem.*, 1995, **117**, 1-19. b)W. L. Jorgensen, D. S. Maxwell, and J. T.-Rives, *J. Am. Chem. Soc.,* 1996, **118**, 11225–11236. c)G. A. Kaminski, R. A. Friesner, J. T.-Rives, and W. L. Jorgensen, *J. Phys. Chem. B*, 2001, **105**, 6474-6487.

[12] a)L. Martínez, R. Andrade, E. G. Birgin, and J. M. Martínez, *J. Comput. Chem.*, 2009, **30**, 2157-2164. b)J. M. Martínez and L. Martínez, *J. Comput. Chem.*, 2003, **24**, 819-825.

[13] a)W. Humphrey, A. Dalke, and K. Schulten, *J. Mol. Graph.*, 1996, **14**, 33-38. b)S. Nosé, *Mol. Phys.*, 1984, **52**, 255-268.

[14] C. Lu, Z. L. Wang, J. Gao, J. J. Li, L. M. Wei, *Adv Energy Mater* **2024**, 14.

[15] W. Q. Chu, X. Zhang, S. Zhao, M. X. Tang, S. X. Li, S. Q. Liu, H. J. Yu, *Adv Funct Mater* **2024**, 34.

[16] E. Hu, B. E. Jia, W. Nong, C. G. Zhang, B. Zhu, D. S. Wu, J. W. Liu, C. Wu, S. B. Xi, D. Xia, M. S. Zhang, M. F. Ng, A. Sumboja, K. Hippalgaonkar, Q. Y. Yan, *Adv Energy Mater* **2024**, 14.

[17] X. Y. Zhang, R. Wang, Z. X. Liu, Q. W. Ma, H. B. Li, Y. Y. Liu, J. N. Hao, S. L. Zhang, J. F. Mao, C. F. Zhang, *Adv Energy Mater* **2024**, 14.

[18] R. Q. Tao, H. W. Fu, C. T. Gao, L. Fan, E. R. Xie, W. Lyu, J. Zhou, B. A. Lu, *Adv Funct Mater* **2023**, 33.

[19] B. E. Jia, E. R. Hu, Z. Y. Hu, J. J. Liew, Z. J. Hong, Y. Q. Guo, M. Srinivasan, Q. Zhu, J. W. Xu, J. Chen, H. G. Pan, Q. Y. Yan, *Energy Storage Mater* **2024**, 65.

[20] D. Y. Wang, E. H. Hu, G. Wu, H. Choo, C. Franke, B. E. Jia, J. X. Song, A. Sumboja, I. T. Anggraningrum, A. Z. Syahrial, Q. Zhu, M. F. Ng, T. Li, Q. Y. Yan, *Angew Chem Int Edit* **2025**.

[21] Z. Zhao, Z. Zhang, W. Wang, T. Xu, X. Yu, *Angew Chem Int Edit* **2025**, 64.

[22] C. Lu, Z. L. Wang, Y. Zhang, G. Tang, Y. Wang, X. Guo, J. J. Li, L. M. Wei, *Nano Energy* **2024**, 120.

[23] Q. Ran, H. Shi, H. Meng, S. P. Zeng, W. B. Wan, W. Zhang, Z. Wen, X. Y. Lang, Q. Jiang, *Nat Commun* **2022**, 13.
